# Microbiota of pest insect *Nezara viridula* mediate detoxification and plant defense repression

**DOI:** 10.1093/ismejo/wrae097

**Published:** 2024-06-05

**Authors:** Silvia Coolen, Magda A Rogowska-van der Molen, Ineke Kwakernaak, Johan A van Pelt, Jelle L Postma, Theo van Alen, Robert S Jansen, Cornelia U Welte

**Affiliations:** Department of Microbiology, Radboud Institute for Biological and Environmental Sciences (RIBES), Radboud University, Heyendaalseweg 135, 6525 AJ Nijmegen, The Netherlands; Translational Plant Biology, Department of Biology, Utrecht University, Padualaan 8, 3584 CH Utrecht, The Netherlands; Department of Microbiology, Radboud Institute for Biological and Environmental Sciences (RIBES), Radboud University, Heyendaalseweg 135, 6525 AJ Nijmegen, The Netherlands; Department of Microbiology, Radboud Institute for Biological and Environmental Sciences (RIBES), Radboud University, Heyendaalseweg 135, 6525 AJ Nijmegen, The Netherlands; Plant-Microbe Interactions, Department of Biology, Utrecht University, Padualaan 8, 3584 CH Utrecht, The Netherlands; Department of General Instrumentation, Faculty of Science, Radboud University, Heyendaalseweg 135, 6525 AJ Nijmegen, The Netherlands; Department of Microbiology, Radboud Institute for Biological and Environmental Sciences (RIBES), Radboud University, Heyendaalseweg 135, 6525 AJ Nijmegen, The Netherlands; Department of Microbiology, Radboud Institute for Biological and Environmental Sciences (RIBES), Radboud University, Heyendaalseweg 135, 6525 AJ Nijmegen, The Netherlands; Department of Microbiology, Radboud Institute for Biological and Environmental Sciences (RIBES), Radboud University, Heyendaalseweg 135, 6525 AJ Nijmegen, The Netherlands

**Keywords:** Arabidopsis thaliana, detoxification, insect–microbe–plant interactions, plant defenses, plant metabolites

## Abstract

The Southern green shield bug, *Nezara viridula*, is an invasive piercing and sucking pest insect that feeds on crop plants and poses a threat to global food production. Given that insects are known to live in a close relationship with microorganisms, our study provides insights into the community composition and function of the *N. viridula*-associated microbiota and its effect on host–plant interactions. We discovered that *N. viridula* hosts both vertically and horizontally transmitted microbiota throughout different developmental stages and their salivary glands harbor a thriving microbial community that is transmitted to the plant while feeding. The *N. viridula* microbiota was shown to aid its host with the detoxification of a plant metabolite, namely 3-nitropropionic acid, and repression of host plant defenses. Our results demonstrate that the *N. viridula*-associated microbiota plays an important role in interactions between insects and plants and could therefore be considered a valuable target for the development of sustainable pest control strategies.

## Introduction

Pest insects are among the major threats to global food production and cause significant crop losses [1–4]. Despite current pest management strategies, a third of the annually produced crops are lost due to pest insects and insect-transmitted plant diseases, and climate change is predicted to further increase these losses [5–9]. In addition, there is an increasing need for reducing pesticide usage to relieve environmental pollution and negative effects on non-target organisms. Together with a rapidly increasing human population, serious problems for food security are expected. Therefore, expanding our knowledge of how insects cope with plant defenses is essential for the development of sustainable pest control strategies [10, 11].

To ward off insects, plants make use of structural barriers (e.g. trichomes) and chemical defenses (e.g. toxic glycosides) that are constitutively present or induced upon recognition of potential threats. In the case of herbivorous insects, the induction of attacker-specific plant defenses is triggered within seconds after wounding, leading to the production and accumulation of phytohormones and toxic secondary plant metabolites [12]. Plant defensive phytohormones salicylic acid (SA) and jasmonic acid (JA) participate in defenses against biotic threats, often acting antagonistically [13]. Piercing and sucking insects, such as those from the order of Hemiptera, usually induce either one or both phytohormones resulting in the expression of downstream signaling genes such as pathogenesis-related 1 (*PR-1*) in the case of SA, and lipoxygenase 2 (*LOX2*) and myeloblastosis (*MYB*) transcription factors (e.g. *MYB28*) in the case of JA [14–20]. Defense signaling eventually leads to the production of secondary metabolite chemical defenses of which thousands are known, covering alkaloids, glucosinolates, polyphenols, and terpenes [21–24].

Insects have coevolved with their host plants and harbor a wealth of adaptations that allow them to feed on plants with specialized defense mechanisms involving secondary plant metabolites [25–28]. Even though insects themselves adapted to coping with toxic compounds, they often acquire symbiotic microorganisms that aid in breaking down toxic plant metabolites through detoxifying symbiosis [4, 24, 29–34].

The Southern green shield bug, *N. viridula* (Pentatomidae: Hemiptera), is an invasive piercing and sucking pest insect that feeds on plant sap of plants, including crop species from the cruciferous, solanaceous, and fabaceous plant families that represent major food crops (e.g. tomato, beans [35]). As in other shield bugs, *N. viridula* has crypts in the M4 section of the midgut that carry species-specific symbionts that are vertically transmitted via egg surface contamination to the offspring [36, 37]. Geerinck *et al.* [38] revealed that the egg-associated community of *N. viridula* consists of mainly Gammaproteobacteria and some Bacilli, specifically a *Pantoea*-like symbiont, *Sodalis* sp., *Serratia* sp., *Niallia* sp., *Staphylococcus* sp., and *Bacillus* sp. strains and a study on adult *N. viridula* showed the dominance of *Pantoea* sp.*, Yokenella* sp.*,* and *Enterococcus* sp. in the midgut [32]. *N. viridula* microbiota has been hypothesized to facilitate detoxification and repression of plant defenses, because insect-associated microbes transmitted while feeding causes vein necrosis in soybean seedlings [32, 39]. Likewise, other insects have been described to transmit their microbiota and redirect plant defenses to their benefit [40]. In the case of the Colorado potato beetle (*Leptinotarsa decemlineata*), the transmission of microbiota during feeding represses plant JA-defenses directed against chewing insects. This repression is caused by microbial SA induction that represses the plant’s JA-defensive pathway via crosstalk [41–43].

To further explore the involvement of insect-associated microorganisms in insect–plant interactions, we studied *N. viridula-*associated microbiota*.* The objective of this study is to characterize *N. viridula* core microbiota across different developmental stages and assess whether it supports plant defense repression and detoxification to the benefit of its host. To this end, we performed 16S rRNA gene and metagenome sequencing, microscopy of insect tissues, bacterial isolation, detoxification assays, and plant infection experiments, revealing that the *N. viridula* microbiota has a profound impact on plant–insect interactions.

## Materials & methods

### Insect collection and rearing

*N. viridula* (42, second and fourth instar nymphs) shield bugs were collected at Romeinenweerd in Venlo (Netherlands, 51.348028, 6.128802), on creeping thistle (*Cirsium arvense, Asteraceae*) on 5 July 2019. Insects were transferred to greenhouse insect-rearing facilities with normal daylight and an additional 16-h photoperiod with artificial light. Insects were allowed to feed on black mustard (*Brassica nigra*), black nightshade (*Solanum nigrum*)*,* crown vetch (*Securigera varia*) and seeds of soybean (*Glycine max*), sunflower (*Helianthus annuus*), and brown mustard (*Brassica juncea*; Supporting Information Methods; Supporting Information Fig. S1).

### Insect dissection

To determine the core microbiota of *N. viridula* with 16S rRNA gene amplicon sequencing, eggs and whole first until fourth instars were used. From the fifth instar onward, gut and salivary glands were dissected from insects and used either combined (for fifth instars) or separated for further analysis (performed in biological triplicate *n* = 3; an overview of the type of specimen used can be found in Supporting Information Fig. S1, Table S1). Insects were dissected directly after submersion in 70% ethanol for 1 min after which movement stopped. Dissection was performed under nonsterile conditions using a stereomicroscope, scalpel, scissor, and forceps. Separation of the complete gut system from the insect body was performed in phosphate-buffered saline solution (PBS; 137-mM NaCl, 2.7-mM KCl, 10-mM Na_2_HPO_4_, 1.8-mM KH_2_PO_4_, pH 7.4) to prevent rupture of the tissue. Both salivary glands and complete gut systems were disrupted by vigorously pipetting or vortexing in lysis buffer for DNA isolation or PBS for culturing and isolation.

### Saliva, frass, egg, and phyllosphere collection

*N. viridula* saliva was collected via a modified method as previously described [44]. Briefly, an artificial feeding solution was put onto a surface-sterilized watch glass, covered with parafilm allowing adult insect to feed exclusively on it for 3 days. Afterward, the solution was plated on Luria–Bertani (LB) agar (0.5% peptone, 0.3% yeast extract, 0.5% NaCl, and 1.5% agar; Supporting Information Methods). Insect frass was collected from adult insects by pipetting deposited frass droplets directly into an Eppendorf tube (Supporting Information Fig. S1). Sterilized egg clusters (collected from black nightshade plants) were obtained with a 70% ethanol wash for 30 s and left to dry before DNA isolation. Phyllosphere was collected by 30-min PBS wash of plant material. For detailed instructions, see the Supporting Information Methods.

### Pure culture isolation and culturing of insect-associated microorganisms

Gut and salivary gland suspensions, saliva (i.e. feeding solution) and frass samples were diluted (10, 100, 1000, and 10 000 times) in PBS for CFU determination and plated on either LB or mannitol agar (2.5% *n*-mannitol, 0.5% yeast extract, 0.3% peptone, 1.5% agar) plates (Supporting Information Fig. S2). Culturing was performed under oxic conditions because anoxic culturing led to comparable results. *Pantoea* sp. was isolated from *N. viridula* gut systems on mannitol and LB agar after 2 days of incubation. *Serratia* sp. was isolated from frass with an overnight culture on LB agar. *Sodalis* sp. was isolated on LB agar from salivary glands samples and after 18-day growth at room temperature. Yeast was isolated from salivary glands by culturing on LB agar for 5 days. All isolated species were transferred to new LB agar plates at least six times before they were considered axenic. Bacterial density was determined with microscopy, using a Bürker–Türk counting chamber, and photometrically using a Cary 60 UV-Vis (Agilent) at 600 nm.

### DNA isolation

Isolation of DNA for 16S rRNA gene and metagenome sequencing was performed using a DNeasy PowerSoil kit (QIAGEN, the Netherlands) including the optional heating step of the samples at 70°C for 10 min in the Powerbead tubes. Tissue was directly put into the lysis buffer in the Powerbead tubes and tubes were vortexed for 10 min at 50 Hz using a TissueLyser LT (QIAGEN). DNA was eluted in 50-μl autoclaved ultrapure demineralized water and yield was measured with a Nanodrop1000 Spectrophotometer and a Qubit dsDNA HS assay kit (Thermo Fisher Scientific Inc., Waltham, USA). For detailed instructions, see the Supporting Information Methods.

### 16S rRNA gene amplicon and metagenomic sequencing

16S rRNA gene amplicon sequencing (V3-V4 regions) was performed by Macrogen (Netherlands) with *Bac341F* and *Bac806R* primers [45, 46] using an MiSeq sequencer (Illumina, San Diego, California, USA; Supporting Information Table S2). Paired-end (2× 301 bp) reads libraries were prepared with the Herculase II Fusion DNA Polymerase Nextera XT Index Kit V2 (Illumina). 16S rRNA gene amplicon data were analyzed via our 16S rRNA amplicon sequencing pipeline. Average number of reads per sample category can be found in Supplementary Fig. S3A.

Identification of pure cultures by 16S rRNA gene amplicon Sanger sequencing was performed by Baseclear (Netherlands) using the *Bac341F* and *Bac806R* primers and additional 18S rRNA *EUKA21F* and *EUKB1791R* primers [47]; Supporting Information Table S2). Raw Sanger sequencing data were analyzed using the Chromas software with a subsequent sequence alignment of forward and reverse reads (excluding 18S rRNA reads that did not overlap) using EMBOSS Needle Alignment and an NCBI nucleotide megablast to determine the identity of the microbial isolates.

Template DNA extracted from guts and salivary glands was used for metagenome sequencing to obtain metagenome-assembled genomes (MAGs) of the *N. viridula* core microbiota. DNA libraries were prepared with a Nextera XT Library Preparation Kit (Illumina) according to the manufacturer's instructions. The libraries were checked for quality and size distribution using an Agilent 2100 Bioanalyzer and a High Sensitivity DNA kit (Agilent Technologies, Santa Clara, California, USA). Quantitation of the libraries was performed with a Qubit dsDNA HS assay kit. Paired-end sequencing (2× 300 bp) was performed using an MiSeq sequencer (Illumina) and a MiSeq Reagent Kit v3 (Illumina), according to the manufacturer's protocol. The quality of Illumina paired-end genomic sequencing data were assessed according to our analysis pipeline (Supplementary Methods). Out of 78 373 844 reads, 64 920 532 passed filtering and were used for downstream analyses.

### 16S rRNA gene amplicon sequencing analysis

16S rRNA gene amplicon data was analyzed with R using the “DADA2” pipeline and the “SILVA” database (v138) for assigning taxonomy [48]. Raw forward reads were trimmed by removing the eight nucleotides from the 5’end, removing 40 nucleotides from the 3’end, an additional 3′ end quality threshold of 20 and an average quality threshold of 20 using “FastqCleaner” [49], leaving 2 984 222 reads after removal of plant chlorophyll sequences. The total number of microbial reads that exceeded >10 000 reads in all insect-related samples (excluding plant samples) were considered the core microbiome, covering 89% (2 650 717) of all reads. Relative abundance within each three biological replicates was calculated as a percentage of the total number of reads (Supplemental Fig. S3 and Tables S2 and S3). A heatmap of the average abundance of three biological replicates was produced using the “R” “pheatmap” package. The Shannon index for all amplicon sequence variants (ASVs) and for the core set of microbiota was calculated accordingly [50].

### Metagenome data analysis

The quality of Illumina paired-end genomic sequencing data was assessed according to our analysis pipeline using FASTQC 0.11.8 (“FastQC,” 2015) before and after quality processing. Quality trimming, adapter removal, and contaminant filtering of reads were performed using “BBDuk” (BBTools 38.75; [51]. Trimmed reads were coassembled *de novo* using “MEGAHIT” 1.2.9; [52]. MEGAHIT assembled the genome using k-mer sizes of 21, 29, 39, 59, 79, 99, 119, and 141. Reads were mapped back to the genomes using separately “BBMAP” 38.75 (default settings), “Bowtie” 2 2.3.5, or Burrows–Wheeler Aligner (“BWA MEM”) 0.1.17 [51, 53]. The sequencing mapping files were handled and converted using “SAMtools” 1.10 [54]. Genome binning was performed for contigs >1500 bp using four binning algorithms: “BinSanity” 0.3.1 [55], “CONCOCT” 1.1.0 [56], “MaxBin2” 2.2.7 [57], and “MetaBAT” 2 2.15 [58] using default settings. The bin sets were supplied to “DAS Tool” 1.1.2 [59] for consensus binning to obtain the final optimized bins. Out of 78 373 844 reads, 64 920 532 passed filtering. The quality of the generated bins was assessed through a single-copy marker gene analysis with “CheckM” 1.1.2 [60]. Taxonomic assignment for the trimmed sequencing reads and MAGs was performed with Kaiju [61]. The metagenomes were automatically annotated with “Prokka” 1.13.4 [62] and the “KEGG” database was used to explore and reconstruct metabolic pathways [63]. Illumina raw sequence reads were subjected to the “phyloFlash” pipeline 3.4 [64] to reconstruct the phylogenetic composition. A heatmap was constructed in R 4.2.1 [65], using the “heatmap.2” package. The metabolic potential of *N. viridula*-associated microbiota was studied using KEGG and “KAAS” (Supplemental Tables S5 and S6; [63, 66].

### Fluorescence *in situ* hybridization

Isolated gut and salivary glands were hybridized with a fluorescein-labeled general bacterial probe (Eub-mix [67, 68]) and a Cy5-labeled Gammaproteobacterial probe (GAM42A [69]) along with a GAM42A competitor probe. An additional Cy3-labeled probe specific for *Sodalis* sp. was used for targeted detection (Sod1238R [70]). Samples were visualized using laser scanning microscopy. For a detailed protocol, see Supporting Information Methods.

### 3-nitropropionic acid degradation assay

*Serratia* sp. was pre-grown in oxic batch cultivation (*n* = 2) for 24 h (30°C, 200 rpm) in M9 mineral salt medium (33.7-mM Na_2_HPO_4_, 22-mM KH_2_PO_4_, 8.55-mM NaCl, 9.35-mM NH_4_Cl, 4-mM glucose, 1-mM MgSO_4_, 0.1-mM CaCl_2_, Thauer vitamin mixture [71], trace elements [72], pH 7.2) until OD_600_ of 1.00 ± 0.05. Hereafter, 100 μM of 3-nitropropionic acid (NPA) was supplemented to the preculture. For determination of NPA, nitrite, and nitrate concentrations, 1-ml samples were collected at 0, 2, 4, 6, and 24 h (*n* = 2). The supernatant was collected by centrifugation at 20 000 × *g* for 3 min and was stored at −20°C until high-performance liquid chromatography (HPLC) analysis, and nitrite and nitrate determination (Supporting Information Methods).

### Quantification of NPA, nitrite, and nitrate

High-performance liquid chromatography (HPLC) was used to determine NPA concentrations. Using an Agilent 1100 system equipped with a diode array detector and a Merck C-18 column (Lichrospher 100 RP-18 end-capped [5 μm] column, 250 mm × 4.6 mm), isocratic analysis was performed with 100% 0.1% ortho-phosphoric acid in water with a flow rate of 1.2 ml min^−1^. Before the analysis, 200 μl of supernatant was acidified with 25-μl 1M sulfuric acid, and 100 μl of the sample was injected. NPA was measured at 210 nm with a retention time of 5.2 min.

To determine nitrite (NO_2_^−^) and nitrate (NO_3_^−^) concentrations a “Griess”-reagent assay was used with standard curves of NaNO_3_ and NaNO_2_. The supernatant (100 μl) was transferred to a 96-well plate containing 100 μl of Griess reagent (50 μl of reagent A: 1% (w/v) sulfanilic acid in 1M HCl and 50 μl of reagent B: 0.1% (w/v) naphthyl ethylene diamine dihydrochloride in demineralized water). The plate was incubated for 10 min at room temperature, and absorbance for nitrite measurement was measured at 540 nm with a microplate reader (SpectraMax 190, Molecular Devices, San Jose, California, United States). Next, 27 μl of VCl_3_ (10 mg ml^−1^ in 1M HCl) was added to the sample to reduce nitrate to nitrite. Subsequently, the 96-well plate was incubated in the dark at 60°C for 30 min, whereafter absorbance was measured at 540 nm.

### *Arabidopsis* inoculation and insect feeding assay

One leaf per mature 5-week-old *Arabidopsisthaliana *plant (Supporting Information Methods) was inoculated with a 5-μl droplet of 1 × 10^8^ CFU/ml bacterial suspension in 10-mM MgSO_4_, followed by a puncture with a sterile 0.4-mm needle through the inoculum into the leaf. Per treatment, three to six plants were inoculated or exposed to insect feeding. After 24 and 72 h, plant leaves were harvested and directly put into liquid nitrogen after which they were stored at −70°C for RNA extraction. Each biological replicate consisted of one to two local (inoculated or feeding damage) leaves from different plants.

### RNA extraction and cDNA synthesis

Plant RNA extractions were performed using a RNeasy Plant Minikit (Qiagen) according to the manufacturer, with the alteration that frozen plant material was ground with a micropestle directly in the extraction buffer. ~150 ng/μl of RNA per sample was used for cDNA synthesis using a QuantiTect Reverse Transcription Kit (Qiagen) according to the manufacturer’s protocol with the prolonged incubation. RNA and cDNA qualities were determined with a Nanodrop.

### *N. viridula* absolute microbial abundance

Real-time quantitative PCR (RT-qPCR) was performed to measure the absolute abundance of bacteria within samples. To quantify all bacteria, 16S rRNA gene primer pair *Bac341F* and *Bac806R*, amplifying V3-V4 regions was used. To determine the abundance of *Pantoea* sp., *Sodalis* sp., *Serratia* sp., and *Commensalibacter* sp., specific primer sets targeting single-copy *groL* and *rpoB* marker genes were designed (*PantF, PantR*. *SodF, SodR, SerF, SerR ComF,* and *ComR*) based on the obtained MAGs from the metagenome analysis (Supporting Information Table S2). RT-qPCR was performed using a pipeline described in detail under the Supporting Information Methods.

### Real-time quantitative PCR of plant cDNA

To determine the relative expression of both plant SA- and JA-defense pathways and the specific activity of the aliphatic glucosinolate secondary metabolite pathway, *A. thaliana* gene primers of *PR-1* (*At2g14610*), *LOX2* (*At3g45140*), and *MYB28* (*At5g61420*) were used (Supporting Information Table S2; [14, 16, 17, 73]), comparing gene expression with the 2^-ΔΔCT^ method to the housekeeping gene *PP2AA3* [74, 75]. For detailed RT-qPCR methods, see the Supporting Information Methods.

### Statistical analyses

For plant defense repression analyses, Student’s *t*-test was performed to identify significant differences between mock and treatment (^*^P ≤ .05, ^*^^*^P ≤ .01, ^*^^*^^*^P ≤ .001).

## Results

### Experimental approach

**Figure 1:**
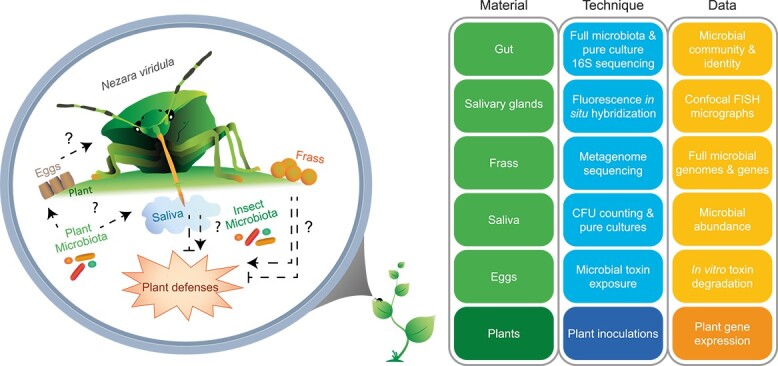
Experimental approach. An overview showing the experimental setup to identify *N. viridula* core microbiota throughout different developmental stages and subsequently unravel its role in insect–plant interactions. In the right panel, the study materials (Supporting Information Fig. S1), techniques used, and eventually obtained data are depicted.

To characterize the core microbiota of *N. viridula* and subsequently unravel its role in insect–plant interactions we compared host plant leaf microbiota with insect eggs, gut systems, salivary glands, saliva, and frass samples using 16S rRNA gene amplicon sequencing (Fig. 1, Supporting Information Fig. S3). Fluorescence *in situ* hybridization (FISH) was performed to visually localize *N. viridula* symbionts. Metagenome analysis was performed on adult insect gut systems and salivary glands to unravel the microbial metabolic potential. *Nezara viridula-*associated microbes were isolated and tested *in vitro* for degradation of plant toxins. Lastly, plant inoculations using isolated pure cultures were performed to determine the effect of *N. viridula* microbiota on host plant defenses.

### *N. viridula* core microbiota consists of two main symbionts

**Figure 2:**
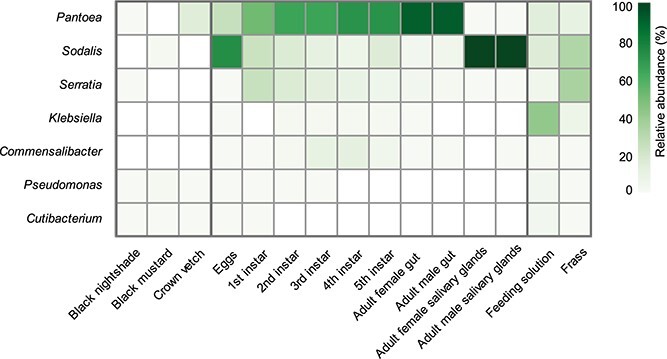
Microbiota of *N. viridula* over different developmental stages. Heatmap showing the relative abundance (in percentage) of the core genera (exceeding >10 000 reads in all insect-related samples, covering 89% (2650717) of all reads) within each sample type consisting of three biological replicates for *N. viridula* samples and one replicate for host plant samples (39 samples in total; Supporting Information Table S1).

The microbiota of *N. viridula* was determined by 16S rRNA gene profiling of all developmental stages. Single plant phyllosphere samples of black nightshade, black mustard, and crown vetch were taken along as a background for transient microbiota from ingested food. Of all *N. viridula* microbiota, the most abundant reads were assigned to the genus *Pantoea* followed by the genera *Sodalis*, *Serratia*, *Klebsiella*, *Commensalibacter*, *Pseudomonas*, and *Cutibacterium* (Fig. 2, Supporting Information Figs S3 and S4A, and Tables S3 and S4). Plant material contained minor numbers of bacterial reads belonging to the genera found highly abundant in *N. viridula*; the high abundance of *Pantoea* sp. and *Sodalis* sp. in surface-sterilized eggs is particularly suggestive of vertical transmission as the analyzed eggs were collected from black nightshade that had only 0.1% *Pantoea* sp. and no *Sodalis* sp. in their phyllosphere samples (Supplemental Fig. S4B). This indicates that transient microbiota ingested via plant material is not responsible for the high numbers of bacteria found in *N. viridula*.

Alpha diversity throughout the different developmental stages showed a steep and significant increase from egg to third instar, indicating that young insects obtained diverse microorganisms from their environment. From the 4th instar onward, a significant alpha diversity decline is seen that can be explained by the fact that insect guts and salivary glands were dissected from these larger animals, whereas younger instars were used as a whole. From fourth and fifth instar to adult another significant decrease in alpha diversity is seen, that can partially be explained by the change in sample type, in which the adult gut and salivary glands were separated (Supplemental Fig. S3C, D, F, and G).

**Figure 3:**
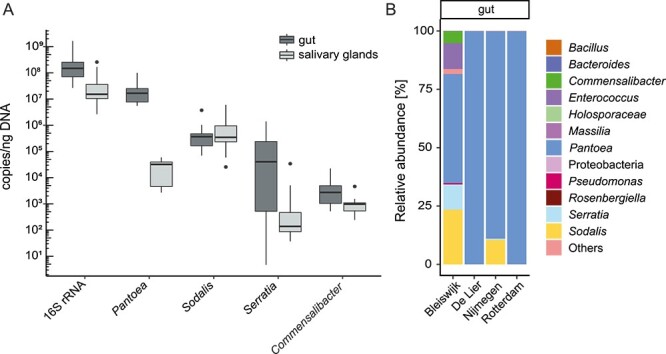
Absolute and relative abundance of *N. viridula* microbiota*.* (A) A box plot showing the mean absolute number of 16S rRNA copies/ng (logarithmic scale) of 11 individual adult insects using RT-qPCR. 16S rRNA from either *Pantoea*, *Sodalis*, *Serratia,* or *Commensalibacter* was used to determine the number of copies compared to genera-specific single-copy genes *gro*L and *rpo*B. On the *x*-axis, the total number of 16S rRNA copies in the gut and salivary glands is shown with subsequent copy numbers for each genus. Error bars represent standard deviations and dots represent outliers. (B) Core bacterial community composition of the gut based on 16S rRNA gene amplicon sequencing. The samples were compared between *N. viridula* adult insects obtained from four geographical locations in the Netherlands. Ten guts and 10 pairs of salivary glands were dissected, pooled, and sequenced, and the taxonomy was displayed whenever possible at the genus level. Others represent the amplicon sequence variances (ASVs) that average below 1% of all reads.

As 16S rRNA gene amplicon sequencing does not account for the variation in 16S rRNA gene copy numbers, absolute abundances of *Pantoea* sp., *Sodalis* sp., *Serratia* sp.*,* and *Commensalibacter* sp. were quantified in individual *N. viridula* adults, using RT-qPCR comparing 16S rRNA gene abundances with single-copy *gro*L and *rpo*B genes (Fig. 3A). Similar to our amplicon sequencing results, *Pantoea* sp. was the most abundant genus, followed by *Sodalis* sp., *Serratia* sp.*,* and *Commensalibacter* sp. Variation in the absolute abundance of bacterial genera between different insects was minimal, except for *Serratia* sp., indicating the stability of the *N. viridula* microbiota. To confirm that aforementioned species (*Pantoea* sp.*, Sodalis* sp.*, Serratia* sp.*, Commensalibacter* sp.) are part of *N. viridula* core microbiota, 16S rRNA gene profiles obtained from adult insects collected from three other locations in the Netherlands were investigated and found to be in accordance with the microbial profile of our greenhouse-reared *N. viridula* (Fig. 3B). The Bleiswijk sample shows the largest deviation from the other locations, which can be explained by extensive inbreeding of this particular research facility rearing population. Overall, all gut samples taken at different locations showed high abundance of *Pantoea* sp. as found earlier.

To determine *N. viridula* microbiota throughout the different developmental stages, we monitored the changes in the relative abundance of the most abundant bacterial genera. Over 25% of the amplicon reads of *N. viridula* egg clusters belonged to the genus *Pantoea*, whereas *Sodalis* sp. represented over 73% (Fig. 2, Supporting Information Table S3). These numbers suggest that both *Pantoea* sp. and possibly also *Sodalis* sp. are vertically transmitted to *N. viridula* and therefore most likely fulfill essential symbiotic functions to their host. In addition, during the development of *N. viridula* from egg to adult, a clear increase in the relative abundance of *Pantoea* sp. was observed. Although *Sodalis* sp. seemed to decrease during development, it could be found highly abundant in salivary glands, indicating that it serves a different role than *Pantoea* sp., which is predominantly found in the gut system (Figs 2 and 3b). All other genera showed a lower abundance throughout *N. viridula’s* development. Our data demonstrate that *Serratia* sp. is obtained by young first instar animals from the environment, and because its abundance in *N. viridula* is higher than that on plants, it seems *N. viridula* also supports the growth of this microorganism. Eventually, *Serratia* sp. declined throughout the development of *N. viridula*, suggesting it is eliminated via an unknown mechanism or resides in organs that were not analyzed. *Klebsiella* sp. was absent from the first instar and found in low abundance on the second instar onward and is, therefore, most likely acquired from the environment. For *Commensalibacter* sp., a similar lack of developmental pattern is observed with a small increase in abundance during the third and fourth instar stage. *Pseudomonas* sp. and *Cutibacterium* sp. were detected in low abundance in both plants and *N. viridula*, suggesting that these genera are transferred via the environment or feeding. The low abundance of *Pseudomonas* sp. in the phyllosphere is probably due to the sampling of young plants grown under controlled greenhouse conditions. Our analysis also revealed that there is no clear difference in the microbiota profiles of adult males and females. Overall, our 16S rRNA gene sequencing results show that the *N. viridula* core microbiota is limited to two symbionts, namely, *Pantoea* sp. and *Sodalis* sp., and a limited consortium of *Serratia* sp., *Klebsiella* sp.*,* and *Commensalibacter* sp. with yet-unclear relationships to their host.

### *N. viridula* core microbiota is transmitted from insects to plants via frass and saliva

16S rRNA gene profiling of frass revealed that it contained ~10% *Pantoea* sp., 33% *Sodalis* sp., 34% *Serratia* sp.*,* and 6% *Klebsiella* sp. (Fig. 2 and Supporting Information Table S3). Frass plated on LB agar resulted in ~1.8 × 10^8^ CFU/ml within a 24-h incubation at room temperature (Supporting Information Figs S5 and S6). The genera *Sodalis* and *Serratia* were present in a relatively high abundance in frass, even though the gut system of the adult animals contained only low abundances of these bacteria. This could be explained by other organs that potentially harbor these bacteria such as Malpighian tubules, which are the extensions of the distal part of the gut system, that are connected to the hindgut.

In contrast to earlier findings, indicating that *N. viridula* saliva obtained through ice exposure–induced salivation is sterile [76], our collected feeding solution offered to *N. viridula* contained excreted saliva with 13% *Pantoea* sp., 16% *Sodalis* sp., 5% *Serratia* sp. and 42% *Klebsiella* sp. (Fig. 2 and Supporting Information Table S3). Control feeding solution systems that were inaccessible to *N. viridula* remained sterile, suggesting that *N. viridula* transmits its associated microbiota during feeding. Culturing of collected feeding solution resulted in ~1.2 × 10^4^ CFU/ml of a particularly slow-growing microorganism on LB agar, which was later identified as yeast, highly similar to the genera *Wickerhamia* and *Candida* (Supporting Information Fig. S5 and Table S5).

Taken together, 16S rRNA gene amplicon sequencing and culturing revealed that the *N. viridula* core microbiota is limited to several microbial genera of which some are transmitted via feeding, which might have consequences on insect–plant interactions.

### *N. viridula* salivary glands are colonized by Gammaproteobacteria and *Sodalis* sp.

**Figure 4:**
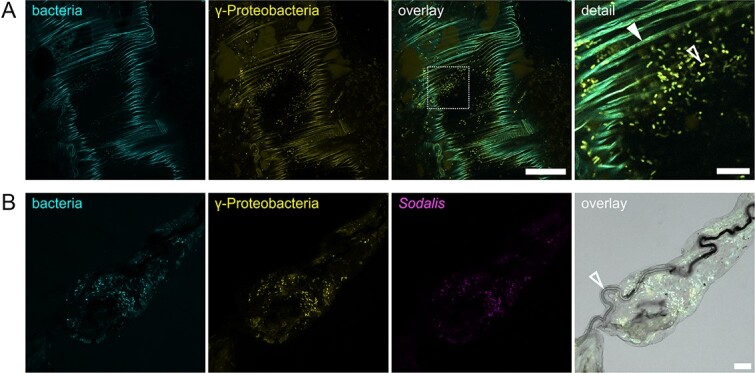
Colonization of *N. viridula* gut and salivary glands by Gammaproteobacteria and *Sodalis*. (A) Confocal micrographs show the cavity of an adult *N. viridula* M4 gut crypt with FISH-stained microbes. Panel "bacteria" shows FISH-probe "Eub-mix" in cyan (Fluos dye). Panel "γ-Proteobacteria" shows FISH-probe "GAM42A" in yellow (Cy5 dye). Panel "overlay" shows the overlay of both channels. Scale bar "overlay" = 50 μm. A dashed square in "overlay" marks the location of the magnified panel "detail". Closed arrowhead points at autofluorescent gut structure. Open arrowhead points at FISH-stained bacterium. Scale bar "detail" = 10 μm. (b) Confocal micrographs show an adult *N. viridula* salivary gland with the salivary duct. Panel "bacteria" shows FISH-probe "Eub-mix" in cyan (Fluos). Panel "γ-Proteobacteria" shows FISH-probe "GAM42A" in yellow (Cy5). Panel "*Sodalis*" shows FISH-probe "Sod1238R" in magenta (Cy3). Panel "overlay" shows the overlay of all three fluorescent channels merged with a transmitted-light brightfield channel. Open arrowhead points at salivary duct. Scale bar = 100 μm.

To visually confirm the localization of *N. viridula*-associated microbiota, we performed FISH on the gut system and salivary glands. We targeted all bacteria with fluorescent probes for Gammaproteobacteria and *Sodalis* sp. and visualized them with confocal laser scanning microscopy. Microscopy images of the gut system confirmed that many rod-shaped Gammaproteobacteria were present in the cavities of the M4 midgut crypts of *N. viridula* (Fig. 4A). Salivary glands of phytophagous shield bugs consist of a principal gland with anterior and posterior lobes, a hilum with a principal salivary duct, the duct of the accessory gland, and the accessory gland [77]. Our analysis revealed that the entire principal gland tissue is colonized with Gammaproteobacteria and *Sodalis* sp. is the dominant member of the community (Fig. 4B and Supporting Information Fig. S7).

### Metagenome analysis reveals a detoxifying potential of *N. viridula* microbiota

**Figure 5:**
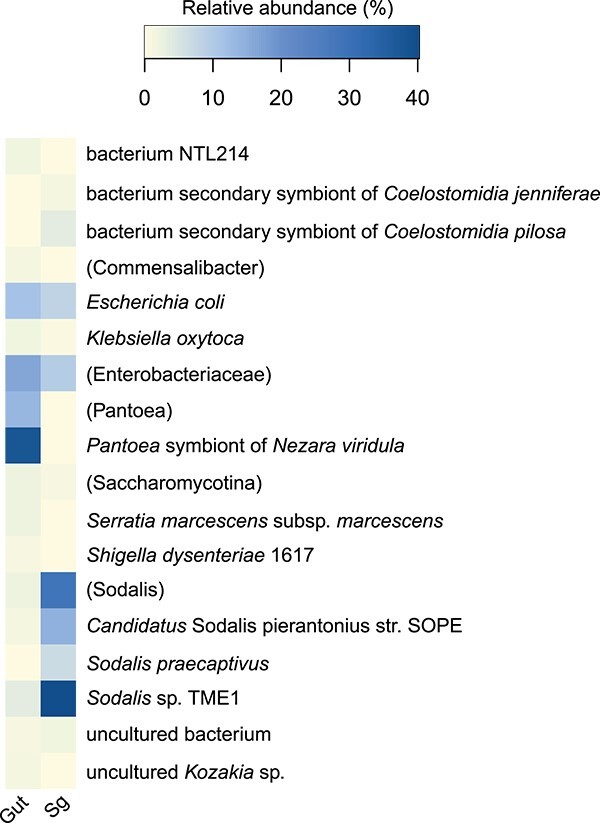
Relative metagenome gut and salivary gland microbial abundance. A heatmap showing the relative metagenome microbial abundance (>1%) in adult *N. viridula* gut systems (gut) and salivary glands (Sg), calculated percentage of reads based on raw reads from the Blood&Tissue DNA isolation kit. When the species could not be assigned, the lowest taxonomic level was shown instead and was indicated with a taxonomic name between the parentheses.

The role and microbial metabolic potential of *N. viridula*-associated microorganisms was analyzed using metagenomics on *N. viridula* gut systems and salivary glands. Similar to the 16S rRNA gene amplicon data, the analysis revealed that *Pantoea* sp. and *Sodalis* sp. were the most abundant species in the gut and salivary glands, respectively (Fig. 5 and Supporting Information Fig. S8). Moreover, we found that *N. viridula* gut and salivary glands harbored several strains of *Pantoea* sp. and *Sodalis* sp. Further metagenomics analysis yielded one metagenome-assembled genome (MAG) for each of the core symbionts *Pantoea* sp., *Sodalis* sp., *Serratia* sp.*,* and *Commensalibacter* sp. (Table 1).

**Table 1:** Overview of the recovered MAGs. The information for each of the MAGs includes bacterial ID, genome size, GC content, completeness, contamination, number of contigs, and the relative abundance assigned based on the percentage of mapped reads. The relative abundance of unmapped reads was assigned to either mitochondrial or chloroplast reads and therefore was left out of the table.

Bacterial IDs (genus)	Genome size (bp)	GC content (%)	Completeness (%)	Contamination (%)	Contigs (*n*)	Relative abundance (%)^a^	
						Gut^b^	Salivary glands^b^
*Commensalibacter*	2 009 072	37.5	95.8	0.0	145	2.5	0
*Pantoea*	1 426 114	40.6	59.3	0.0	12	57	0
*Serratia*	5 249 834	59.8	99.8	0.5	91	9	0
*Sodalis*	4 205 851	56.7	100	0.0	189	7.3	68.5

a The abundance of the mapped reads within a sample.

b Sample isolated using a Blood&Tissue kit.

*Nezara viridula* feeds on nutritious-poor and sugar-rich plant sap and relies on its symbiotic partners to biosynthesize deficient nutrients [37]; therefore, we analyzed the metabolic potential of *N. viridula*-associated microbes in terms of amino acid and vitamin metabolism and the degradation of carbohydrates (Supporting Information Tables S6 and S7). *Pantoea* sp., *Sodalis* sp., and *Serratia* sp. can biosynthesize most essential amino acids and all core microbes harbor partial vitamin B biosynthetic pathways, although none can biosynthesize thiamine or cobalamin. In terms of carbohydrate-degrading properties, *Sodalis* sp. harbors genes to degrade starch, and D-galacturonate (Supporting Information Table S7). A similar degradation potential was observed for *Serratia* sp., yet it lacked the genes to degrade D-galacturonate, a key constituent of pectin. *Commensalibacter* sp. has the metabolic potential to degrade starch and, unlike others, might convert arabinan to L-arabinose and 1,4-beta-D-xylan to D-xylose, which are dominant in woody plants, plant seeds, and root components [78, 79]. *Pantoea* sp. lacked most carbohydrate-degrading enzymes and is therefore likely not involved in the degradation of carbohydrates. In addition, both *Serratia* sp. and *Sodalis* sp. contained chitin degradation genes, which could support insect resistance to fungal pathogens [80].

*Nezara viridula* microbiota could also provide its insect host with unique characteristics that may play a role in establishing symbiotic interactions with insects via cellular invasion, as well as the colonization of plants [81, 82]. We discovered that *Sodalis* sp. encodes a complete bacterial type III secretion system (T3SS, *yscFCJRSTUVNQ*) that is used by bacteria to secrete effector proteins, which can manipulate or even repress plant defenses [83]. The latter can potentially reduce the production of anti-insect feeding compounds and toxic metabolites [84, 85].

Given that insects used in this study were reared on native black nightshade and crown vetch plants that produce toxic α-solanine, α-chaconine, and NPA metabolites, detoxifying symbiosis was investigated. α-Solanine and α-chaconine are two structurally related glycoalkaloids that inhibit cholinesterase activity and disrupt eukaryotic cells and therefore are likely harmful to *N. viridula* [86]. *Serratia* sp.*, Sodalis* sp.*,* and *Commensalibacter* sp. were confirmed to contain genes involved in α-solanine and α-chaconine degradation via β-galactosidase, β-glucosidase, and α-rhamnose isomerase ([87]; Supporting Information Table S7) although none of the isolated strains degraded α-solanine and α-chaconine *in vitro* under the chosen conditions. NPA is known to be toxic to eukaryotes by irreversibly inhibiting an enzyme from the tricarboxylic acid cycle [88]. Detoxifying symbiosis by *N. viridula* symbiont *Serratia* sp. by nitronate monooxygenase (*nmoA*), 3-oxopropanoate dehydrogenase (*bauC*), nitrite reductase (*nasD*), and flavohemoprotein (*hmp*) potentially supports its insect host with NPA degradation. Overall, genetic evidence points toward the possible involvement of *N. viridula* microbiota in the biosynthesis of nutrients, digestion of carbohydrates, suppressing plant defenses, and in detoxifying symbiosis.

### *Serratia* sp. degrades 3-nitropropionic acid

**Figure 6:**
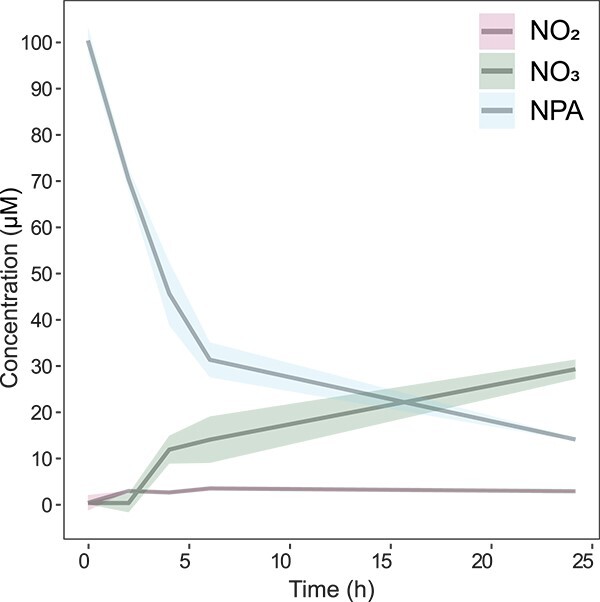
NPA degradation by *N. viridula*-associated *Serratia*. *Serratia* was grown in M9 medium until an OD_600_ of 1.00 ± 0.05. Hereafter, 100 μM of NPA was added, and samples were immediately taken (0 h) and after 2, 4, 6, and 24 h (*n* = 2). NPA (blue) was measured with HPLC and nitrite (NO_2_^−^, red) and nitrate (NO_3_^−^, green) concentrations were determined using a Griess assay. On the *x*-axis, the time is shown in hours (h) and on the *y*-axis NPA concentration (μM). Shaded areas represent standard error.

*Serratia* sp., which harbored detoxifying genes, was cultured with NPA for 24 h to unveil whether *N. viridula* symbionts can degrade toxic plant metabolites. Rapid degradation of NPA was observed for the first 6 h of incubation, after which the rate of degradation decreased (Fig. 6). Simultaneously, nitrate and to a lesser extent nitrite by-product concentrations increased. No nitrate nor nitrite were present in control cultures, confirming that they are by-products of NPA degradation (data not shown). These results demonstrate the ability of gut-associated *Serratia* sp. to degrade the toxic plant metabolite NPA.

### *N. viridula*-associated microbiota repress plant defenses

**Figure 7:**
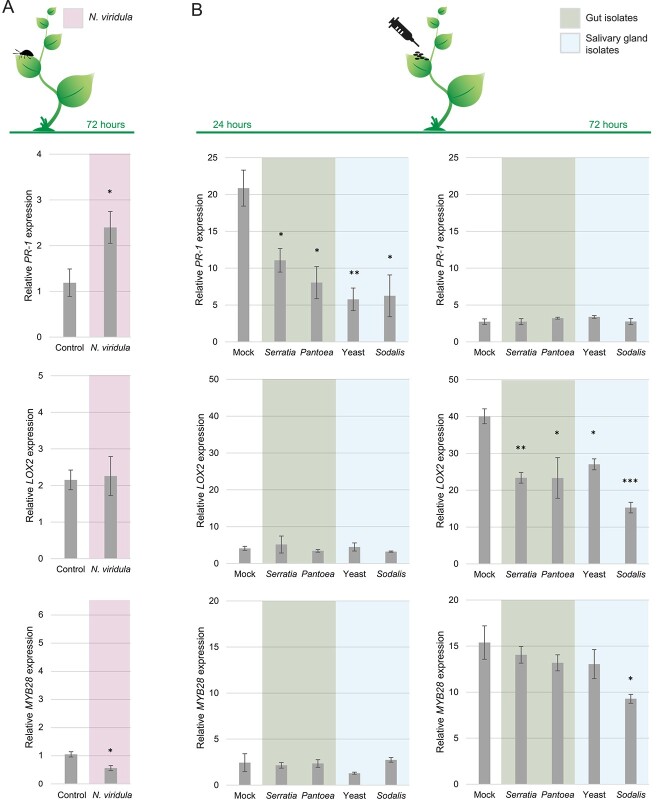
Plant defense repression by *N. viridula*-associated microorganisms. (A) *PR-1*, *LOX2,* and *MYB28* gene expression of local leaves (feeding damage) from 5-week-old *a. thaliana* plants infested by *N. viridula* adults for 72 h and control plants without *N. viridula* (*n* = 3, 6 plants, 9 technical replicates). (B) *PR-1*, *LOX2,* and *MYB28* gene expression of 5-week-old *A. thaliana* plants at 24 and 72 h after pierce-inoculation with a 5-μl droplet of 10-mM MgSO4 (i.e. mock) or 1 × 10^8^ CFU/ml of *Serratia*, *Pantoea*, yeast, or *Sodalis* culture suspension in 10-mM MgSO4 (*n* = 3, 3 plants, 4 technical replicates). Bars represent the average gene expression relative to *PP2AA3*, and error bars represent the standard error of means. Asterisks show Student’s *t*-test significant differences between mock and treatment (^*^P ≤ .05, ^*^^*^P ≤ .01, ^*^^*^^*^P ≤ .001).

To determine whether *N. viridula’*s saliva and frass microbiota, namely *Pantoea* sp., *Serratia* sp., *Sodalis* sp. and yeast sp. could alter plant defenses, they were inoculated on mature *Arabidopsis thaliana* plants (Brassicaceae). The results showed that when *N. viridula* fed on *Arabidopsis* for 72 h, it significantly induced *PR-1* expression and suppressed *MYB28* expression (Fig. 7 and Supporting Information Fig. S9). We also observed that the four tested *N. viridula*-associated microorganisms were able to significantly repress artificial piercing–induced *PR-1* expression at 24 h after inoculation, raising the possibility that they also repress *N. viridula*-induced *PR-1* expression at 72 h of feeding. At 72 h of feeding, no *LOX2* induction was observed. On the contrary, at 72 h, all tested *N. viridula* microbiota repressed artificial piercing-induced *LOX2-*expression. JA-associated defense responses have previously been shown to be induced by *N. viridula* 3 h after feeding [15]. Taken together, the current data suggest that *N. viridula* microbiota repress both *N. viridula*-induced SA and JA pathways. *Sodalis* sp. was the only microorganism that could significantly repress *MYB28* expression at 72 h after inoculation, thereby potentially directly suppressing the plant’s aliphatic glucosinolate defense pathway. In conclusion, our hypothesis that *N. viridula*-associated microbiota support their host by counteracting insect-induced plant defenses was confirmed.

## Discussion

*N. viridula* is a notorious pest insect threatening global food production [35]. Different pest management strategies were shown ineffective, due to *N. viridula*’s fast adaptive abilities, resulting in the proposition of an integrated approach in which a variety of preventive and therapeutic methods are used [89–93]. As most insects rely on symbiotic microbiota for essential tasks, pest management strategies specifically targeting insect microbiota may provide an additional facet in integrated pest management for *N. viridula*. Strikingly, *N. viridula* microbiota is still poorly investigated, leaving untapped opportunities. To this end, we characterized *N. viridula* microbiota throughout their development as well as for their functional potential.

Our study revealed that the core microbiota of *N. viridula* predominantly consisted of *Pantoea* sp., *Sodalis* sp.*, Serratia* sp., and *Commensalibacter* sp. and to a lesser extent other genera such as *Klebsiella* and *Pseudomonas*, accounting for more than 99% of all microbial members. We determined that *Pantoea* sp. and *Sodalis* sp. were possibly vertically transmitted via eggs as they were present from (surface-sterilized) eggs to adult and thus most likely function as obligate symbionts [38, 94]. Another explanation for their continued presence would be the reacquisition from the environment at different developmental stages, which seems unlikely due to their low relative abundance on the host plants. *Pantoea* sp. was previously described in Pentatomidae such as *N. viridula* and elimination resulted in severe fitness defects of the insect host, confirming its obligate symbiotic nature [95, 96]. *Sodalis* sp. was associated with Pentatomidae before [97], and we were able to pinpoint its predominant localization and highly abundant colonization of salivary glands. Moreover, with metagenomic analysis we shed light onto the role and host-specific adaptations and of *N. viridula* core microbiota, such as genome reduction of *Pantoea* sp. and *Commensalibacter* sp. [98–100], presence of T3SS in *Sodalis* sp., which could allow cellular invasion and modulation of plant defenses [81, 82] and the ability of microbiota to biosynthesize amino acids and B vitamin and degrade plant carbohydrates and toxic secondary plant metabolites. Although, *Pantoea* sp. and *Sodalis* sp. seem to be most dominant *N. viridula* symbionts, lower abundant *Serratia* sp.*, Klebsiella* sp.*, Commensalibacter* sp.*, Pseudomonas* sp.*,* and *Cutibacterium* sp. could also benefit its insect host. *Serratia* sp. was previously described as a facultative symbiont of insects that could be obtained via plants, and in this study, we demonstrated its possible participation in detoxifying symbiosis via culturing it with toxic NPA [101]. Even though some *Serratia* species are notorious plant disease–causing organisms that colonize the plant’s phloem [102], no visual disease symptoms were observed in our plant inoculation experiments. Furthermore, *Klebsiella* sp. was detected in symbiotic relations with insects including Mediterranean fruit flies (*Ceratitis capitata*) and showed competitive capacities to pathogenic host gut inhabitants [103, 104]. *Commensalibacter* sp., isolated from the midgut of *Drosophila*, has been demonstrated to protect against gut pathogens and our finding additionally implies its contribution to degradation of α-solanine and α-chaconine [105]. Also, *Pseudomonas* sp. Nvir isolated from *N. viridula* gut could detoxify NPA and therefore possibly benefits its host [88]. Besides bacterial symbionts, we also isolated a yeast species closely related to *Wickerhamia* sp. and *Candida* sp. from salivary glands and although insect-associated yeasts are still poorly studied, a previous study suggested their nutritional support to insects by biosynthesizing amino acids and vitamins [106].

Along with nutritious, protecting and detoxifying properties of *N. viridula-*associated microbes, we found that *N. viridula* transmits bacteria during feeding via saliva that altered plant defenses. *Nezara viridula* induced SA (i.e. *PR-1*) plant defenses, which is in line with previously published results [15], whereas all isolated microorganisms repressed SA defenses early after inoculation. *Nezara viridula* repressed the aliphatic glucosinolate pathway (i.e. *MYB28*) involved in defense against insects and *Sodalis* sp., predominantly located in the salivary glands, was the only microorganism significantly suppressing the latter pathway. This observation along with the metagenomic evidence of T3SS presence further strengthens the hypothesis that *Sodalis* sp. is a key player in repressing the biosynthesis of secondary plant metabolites. Altogether, *N. viridula* microorganisms seem to have a secret agenda in supporting their host with detoxifying and suppressing properties. However, to fully verify that *N. viridula* microbiota mediates detoxification and plant defense repression, eliminating *N. viridula* microbiome would provide the required evidence for microbial involvement in these processes. Nonetheless, to this day eliminating all microbiota remains challenging, because *N. viridula* requires its microbiota for viability and development [30, 37, 107].

In conclusion, our study reveals the developmental and organ-specific dynamics of *N. viridula* core microbiota, revealing the important roles of *Pantoea* sp., and *Sodalis* sp. as obligate symbionts and *Serratia* sp. and *Commensalibacter* sp. as facultative symbionts. *Nezara viridula* symbionts were found to be involved in host plant defense repression, and *Serratia* sp. revealed *in vitro* detoxifying activities on toxic crown vetch metabolite NPA. Our results show the importance of studying tri-trophic interactions between insects, associated microbiota, and host plants to obtain fundamental knowledge on interactions between different organisms that could lead to the development of sustainable pest management strategies.

## Supplementary Material

Final_version_Supplementary_Information_June2024_wrae097

Final_version_Supplementary_Tables_MAY2024_wrae097

## Data Availability

All sequencing data are available under project number PRJEB64167 and submission ERA24747878.
