# Supplementary material for: Microbiota of pest insect Nezara viridula mediate detoxification and plant defense repression
Source: ISME J. 2024 Jun 5;18(1):wrae097. doi: 10.1093/ismejo/wrae097 (PMC11195473; doi:10.1093/ismejo/wrae097)
Supplement: Final_version_Supplementary_Information_June2024_wrae097 [file final_version_supplementary_information_june2024_wrae097.docx]

**Supplementary Information**

**Microbiota of pest insect *Nezara viridula* mediate detoxification and plant defense repression**

Silvia Coolen^1*^, Magda A. Rogowska-van der Molen^1*^, Ineke Kwakernaak^1^, Johan A. van Pelt^2^, Jelle L. Postma^3^, Theo van Alen^1^, Robert S. Jansen^1^, Cornelia U. Welte^1^

^1^Department of Microbiology, Radboud Institute for Biological and Environmental Sciences (RIBES), Radboud University, P.O. Box 9010, 6500 GL Nijmegen, The Netherlands

^2^Plant-Microbe Interactions, Department of Biology, Utrecht University, PO Box 800.56, 3508 TB, Utrecht, The Netherlands.

^3^Department of General Instrumentation, Faculty of Science, Radboud University, Heyendaalseweg 135, 6525AJ Nijmegen, The Netherlands

^*^These authors contributed equally to this work

**Supplementary Methods**

**Plant material and growing conditions**

Seeds of *N. viridula* host plants *Brassica nigra* (black mustard) and *Securigera varia* (crown vetch) were obtained from ‘De Bolderik’ (Netherlands), whereas *Solanum nigrum* (black nightshade) seeds were collected from a field in the Netherlands (52.061638, 6.411724). Seeds were stratified for two days at 4 ºC in water and *S. varia* seeds were scarified beforehand to improve germination. Seeds of all host plants were directly sown into potting soil in the greenhouse with natural daylight supported by 16h of artificial light per day.

*Arabidopsis thaliana* Col-0 seeds were provided by the Plant-Microbe Interactions group from Utrecht University (Netherlands). *A. thaliana* seeds were sown on autoclaved river sand supplemented with half-strength Hoagland solution containing sequestrene (Van Wees et al 2013). Sown seeds were kept moist in an enclosed environment and stratified for two days at 4 ºC, after which they were transferred to a climate chamber at 21 ºC and a 10-h day /14-h night rhythm (150 µmol m^-2^ sec^-1^).

**DNA isolation**

For the metagenome analysis, DNA was extracted from five pooled guts and five sets of salivary glands using two DNA isolation methods to obtain higher sequencing depth and facilitate bioinformatic analyses. Samples were homogenized by brief vortexing in 200 µL of PBS with 2 mm glass beads. Subsequently, the samples were split and even parts were used for DNA extraction using the DNeasy PowerSoil kit and the DNeasy Blood & Tissue kit (QIAGEN), respectively, according to the manufacturer’s instructions with modifications, namely 100 µL of gut or salivary gland suspension instead of soil was bead-beaten on the TissueLyser LT for 2 min at 50 Hz and DNA was eluted with 50 µL Nuclease Free Water (Thermo Fisher Scientific Inc.). Eluted DNA was incubated at 65°C for 5 min and stored at 4°C until sequencing. Quantification of the extracted DNA was performed with a Qubit dsDNA HS assay kit (Thermo Fisher Scientific Inc.).

**Fluorescence *in situ* hybridization**

Isolated gut and salivary glands were washed two times with PBS and incubated with 300 µL PBS and 900 µL paraformaldehyde solution (4% paraformaldehyde in PBS) for 2 hours on ice and samples were washed twice with 500 µL PBS. The fixed tissue samples were stored in a 1:1 (v/v) PBS-ethanol solution at 4°C until further use. Samples were placed on SuperFrost Plus™ adhesion microscope slides (Epredia™, USA) and dehydrated in increasing ethanol concentration (50%, 80%, 100%) for 10 min each. Then the samples were air-dried and incubated for 3 hours at 46 °C with a hybridization buffer (0.9 M NaCl, 0.02M Tris-HCl (pH 8.0), 35% formamide, 0.02% sodium dodecyl sulfate) and specific probes in the humidified chamber. For bacterial detection and identification of specific bacterial groups within the samples, fluorescence in situ hybridization (FISH) using a Fluos-labelled general bacterial probe (Eub-mix; (Amann et al 1990, Daims et al 1999)) and a Cy5-labeled Gammaproteobacterial probe (GAM42A;(Manz et al 1992)) along with a GAM42A competitor probe were employed. In the case of salivary glands, an additional Cy3-labeled probe specific for *Sodalis* was used for targeted detection (Sod1238R;(Koga et al 2013)). A volume of 300 µL of hybridization buffer was used for the gut sample and 50 µL for the salivary glands with a final Cy3 and Cy5-probe concentration of 0.5 pmol µL^-1^ (Sod1238R and GAM42A) and a Fluos-probe concentration of 0.83 pmol µL^-1^ (Eub-mix).

After hybridization, tissue samples were washed in the wash buffer (0.2 M Tris-HCl (pH 8), 0.08 M NaCl, 5 mM EDTA) for 10 min at 48°C, dipped in ice-chilled ultrapure Milli-Q® water, air-dried and embedded in Vectashield without DAPI (4,6-diamidino-2-phenylindole; Vector Laboratories, Burlingame, CA, USA). Images were taken using a confocal laser scanning microscope (Leica SP8X, Mannheim, Germany) and image processing was performed in Fiji (Schindelin et al 2012).

**Saliva, frass, egg, and phyllosphere collection**

To determine if *N. viridula* transfers microbiota into the host plant during feeding, and potentially manipulate plant defenses-signaling, saliva was collected via a modified method described by Tan et al (2016) using a filter-sterilized feeding solution of 20% sugar, 100 mM L-serine, 100 mM methionine and 38.5 mM aspartic acid (pH=7.0, adjusted with NaOH). The feeding solution was put into a surface-sterilized (70% ethanol) watch glass covered with surface-sterilized parafilm using a syringe and 0,4 mm needle (Sterican). *N. viridula* (*n* = 10) was allowed to feed from the feeding solution (~2 mL) for approximately three days after which the solution was collected and plated on Luria-Bertani (LB) agar plates (0.5% peptone, 0.3% yeast extract, 0.5% NaCl and 1.5% agar) and incubated at room temperature (Supplemental Figure S1). A control system with a feeding solution was kept next to the cage for the same period. After collection, part of the samples was plated on LB agar for colony forming unit (CFU/mL) determination and obtaining pure cultures, whereas the remainder was used for DNA isolation.

To determine if insect frass, which is deposited on host plants, influences the interaction between the insect and its host, frass was collected from adult *N. viridula* shield bugs. Shield bugs were picked up from their host plant and allowed to move on a surface-sterilized nitrile glove. Frass was collected by pipetting deposited droplets directly into an Eppendorf tube (Supplemental Figure S1). As a control, a droplet of sterile PBS was put on a surface-sterilized glove. After sampling, part of the collected frass was plated on LB agar for CFU determination, while the remainder was used for obtaining pure cultures and DNA isolation.

To determine the vertical transmission of microbiota through eggs, egg clusters (collected from black nightshade plants) were surface sterilized with 70% ethanol for 30 sec and left to dry before DNA isolation (Supplemental Figure S1). As a control for transient microbes passing through the insect gut system, all three (non-infested) plant species (five grams each) were harvested and washed for 30 minutes in PBS with six one-minute vortex periods (Supplemental Figure S1). Tubes were spun down for 15 min at 3220 rpm and pellets were used for DNA isolation.

***N. viridula* absolute microbial abundance**

Single copies of the 16S rRNA gene, *gro*L, and *rpo*B genes were cloned into a pGEM-T Easy vector (Promega, Netherlands) and used to produce standard curves. RT-qPCR was performed using a C1000 Touch thermocycler with a CFX96 Touch Real-Time PCR detection system (Bio-Rad Laboratories, Netherlands). The 25 µL reaction mix consisted of 12.5 µL PerfeCTa SYBR Green FastMix (Quanta Bio, USA), forward and reverse primers at a final concentration of 0.2 pmol/µL, 1 μL template DNA with a DNA concentration of 2 ng/µL and Nuclease Free Water (Thermo Fisher Scientific). The RT-qPCR program was initiated by denaturation at 95 °C for 3 min, followed by 40 cycles of denaturation at 95 °C for 30 seconds, annealing at 58 °C for 30 seconds, and elongation at 72 °C for 30 seconds. Finally, a melt curve from 65 °C to 95 °C for 5 seconds was used for measuring the specificity of the RT-qPCR amplification. Standard curves ranging from 10^1^ to 10^9^ copies of the 16S rRNA gene, *gro*L, and *rpo*B genes were calculated and used for the determination of absolute abundances. RT-qPCR efficiency was calculated from the standard curve slope, and was accepted if ≥ 90% and ≤ 110%.

**RT-qPCR of plant cDNA**

Equal amounts of cDNA (10 ng/µL) were used for a 4-step RT-qPCR with a Bio RAD CFX96 Real-Time System and using PerfeCTa SYBR® Green FastMix to monitor the synthesis of double-stranded DNA. The following thermal profile was used: 95 °C for 10 min, 40 cycles of 95 °C for 15 s, 60 °C for 1 min followed by 72 °C for 30 s. Melt curves were recorded after cycle 40 by heating from 60 to 95 °C with a ramp speed of 1.0 °C min^-1^. To determine the relative expression of both SA- and JA-defense pathways and the specific activity of the aliphatic glucosinolate secondary metabolite pathway, the 2^-ΔΔCT^ method was used comparing gene expression to the housekeeping gene *PP2AA3* (Pfaffl 2001). *A. thaliana* genes *PR-1* (*At2g14610*), *LOX2* (*At3g45140*), and *MYB28* (*At5g61420*) were included (Abe et al 2008, Beekwilder et al 2008, Hickman et al 2017, Proietti et al 2018). Samples were run in 4-9 technical replicates. Transcript levels were calculated relative to the *A. thaliana* reference gene *PP2AA3* (Czechowski et al 2005); Supplemental Table S2).

**References**

Abe H, Ohnishi J, Narusaka M, Seo S, Narusaka Y, Tsuda S *et al* (2008). Function of jasmonate in response and tolerance of *Arabidopsis* to thrips feeding. Plant Cell Physiol **49:** 68-80.

Amann RI, Binder BJ, Olson RJ, Chisholm SW, Devereux R, Stahl DA (1990). Combination of 16S rRNA-targeted oligonucleotide probes with flow cytometry for analyzing mixed microbial populations. Appl Environ Microbiol **56:** 1919-1925.

Beekwilder J, Van Leeuwen W, Van Dam NM, Bertossi M, Grandi V, Mizzi L *et al* (2008). The impact of the absence of aliphatic glucosinolates on insect herbivory in *Arabidopsis*. PLoS ONE **3:** e2068.

Czechowski T, Stitt M, Altmann T, Udvardi MK, Scheible W-R (2005). Genome-wide identification and testing of superior reference genes for transcript normalization in *Arabidopsis*. Plant Physiol **139:** 5-17.

Daims H, Bruhl A, Amann R, Schleifer KH, Wagner M (1999). The domain-specific probe EUB338 is insufficient for the detection of all Bacteria: development and evaluation of a more comprehensive probe set. Syst Appl Microbiol **22:** 434-444.

Hickman R, Van Verk MC, Van Dijken AJH, Mendes MP, Vroegop-Vos IA, Caarls L *et al* (2017). Architecture and dynamics of the jasmonic acid gene regulatory network. Plant Cell **29:** 2086-2105.

Koga R, Bennett GM, Cryan JR, Moran NA (2013). Evolutionary replacement of obligate symbionts in an ancient and diverse insect lineage. Environ Microbiol **15:** 2073-2081.

Manz W, Amann R, Ludwig W, Wagner M, Schleifer K-H (1992). Phylogenetic oligodeoxynucleotide probes for the major subclasses of proteobacteria: problems and solutions. Syst Appl Microbiol **15:** 593-600.

Pfaffl MW (2001). A new mathematical model for relative quantification in real-time RT-PCR. Nucleic Acids Res **29:** e45.

Proietti S, Caarls L, Coolen S, Van Pelt JA, Van Wees SCM, Pieterse CMJ (2018). Genome-wide association study reveals novel players in defense hormone crosstalk in Arabidopsis. Plant Cell Environ **41:** 2342-2356.

Schindelin J, Arganda-Carreras I, Frise E, Kaynig V, Longair M, Pietzsch T *et al* (2012). Fiji: an open-source platform for biological-image analysis. Nat Methods **9:** 676-682.

Tan X, Xu X, Gao Y, Yang Q, Zhu Y, Wang J *et al* (2016). Levels of salivary enzymes of *Apolygus lucorum* (Hemiptera: Miridae), from 1st instar nymph to adult, and their potential relation to bug feeding. PLoS ONE **11:** e0168848.

Van Wees SCM, Van Pelt JA, Bakker PAHM, Pieterse CMJ (2013). Bioassays for assessing jasmonate-dependent defenses triggered by pathogens, herbivorous Insects, or beneficial rhizobacteria. Meth Mol Biol **1011:** 35-49.

**Supplementary Figures**


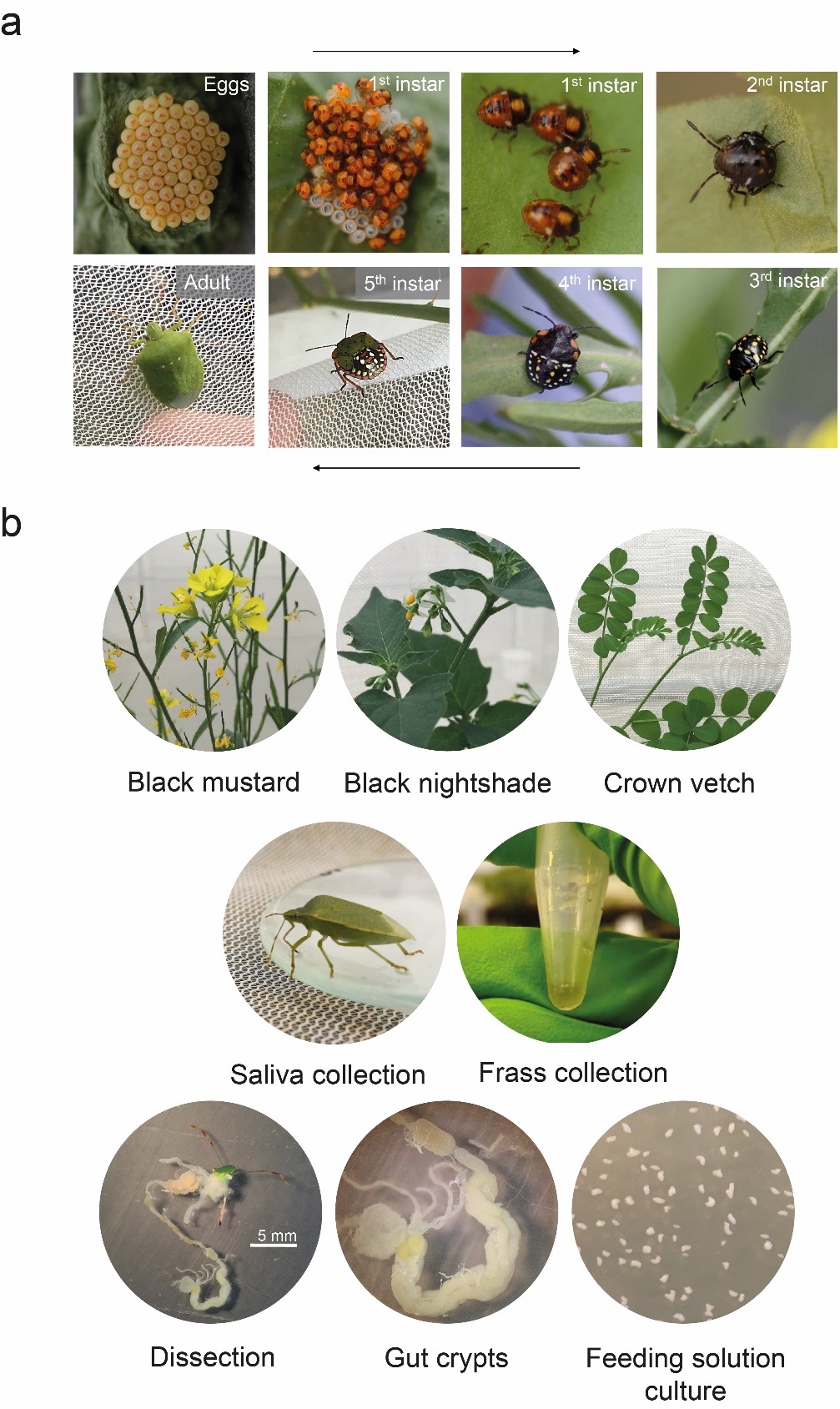


**b**

**a**

**Figure S1. Study material overview.** (**a**) Photographs of all developmental stages, from egg until adult, of *N. viridula* that were used in this study.(**b**) Additional materials and methods used in this study, including plant material of black mustard, black nightshade, and crown vetch, saliva (feeding solution) and frass collections, dissection of *N. viridula* salivary glands and gut system including the midgut crypts and an example of a saliva collection culture showing yeast colonies on LB agar.


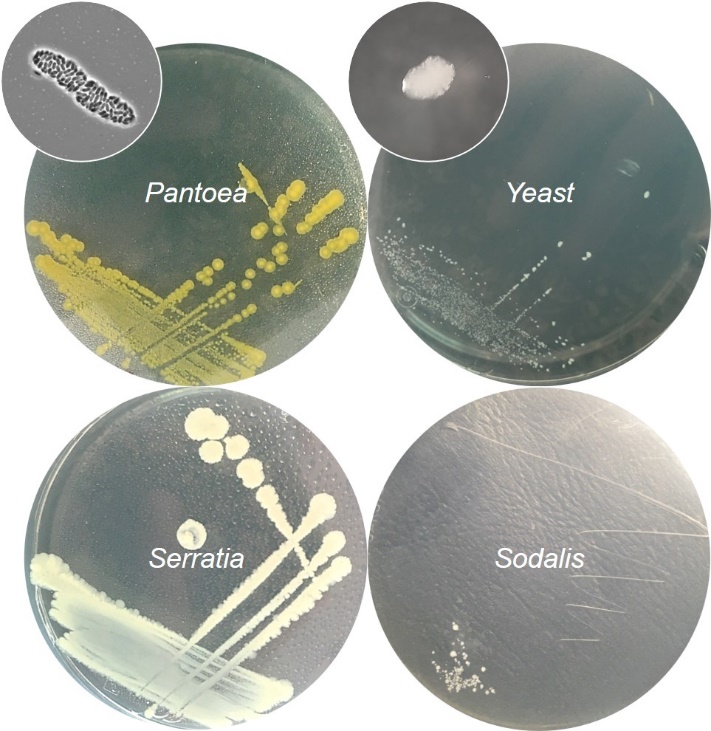


**Figure S2. Isolated microbiota morphology on LB agar plate.** LB agar plates showing isolate colony morphology. In the top left *Pantoea* (3-day culture) forms agglomerations in liquid culture as depicted by the smaller picture. On the top right, Yeast (5-day culture) growing in small irregularly shaped colonies as shown in the magnification of a colony. In the bottom left *Serratia* (2-day culture). On the bottom right *Sodalis*. (3-week culture).

**
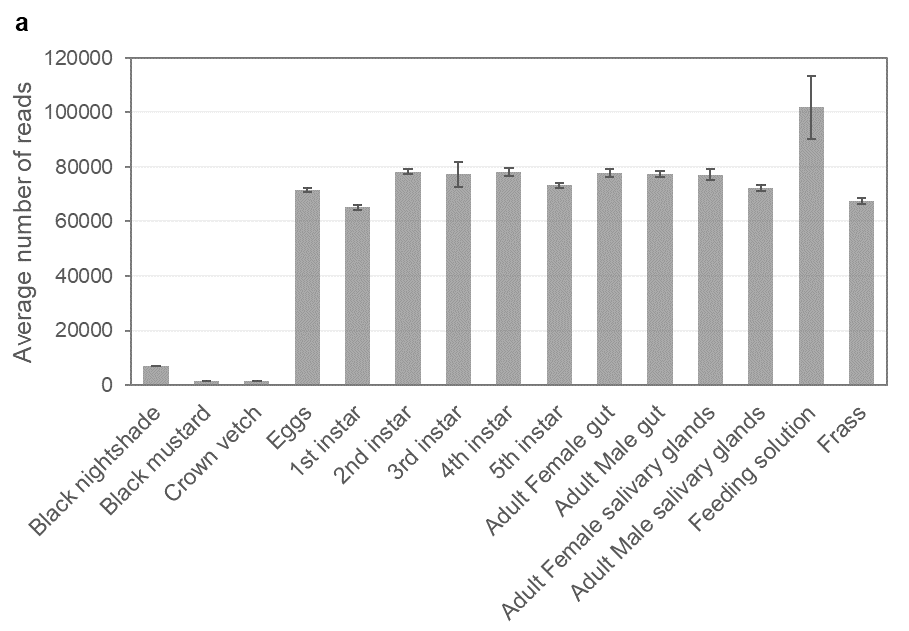

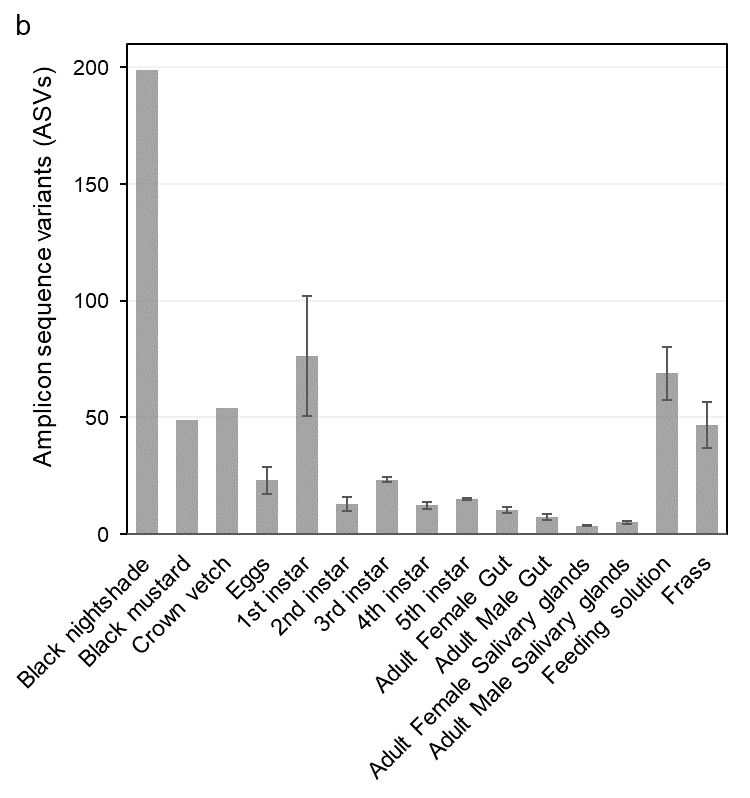
**

**
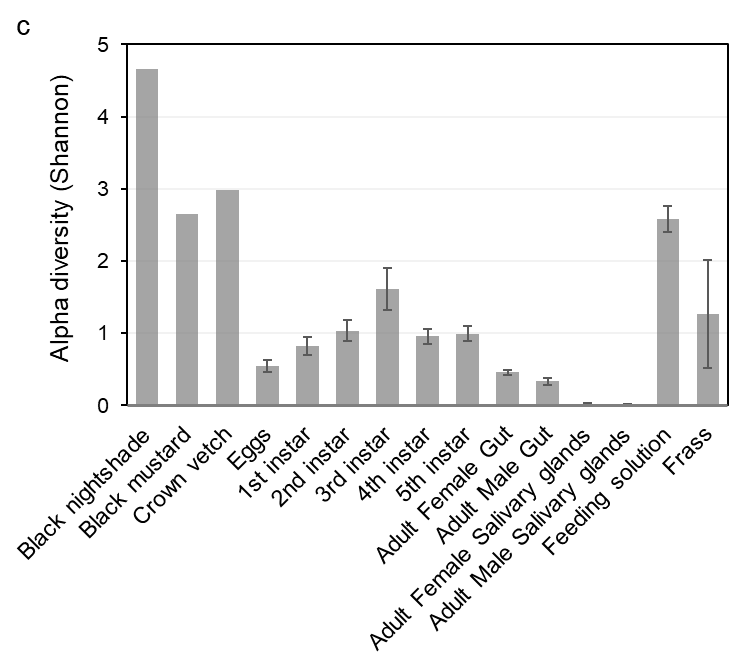

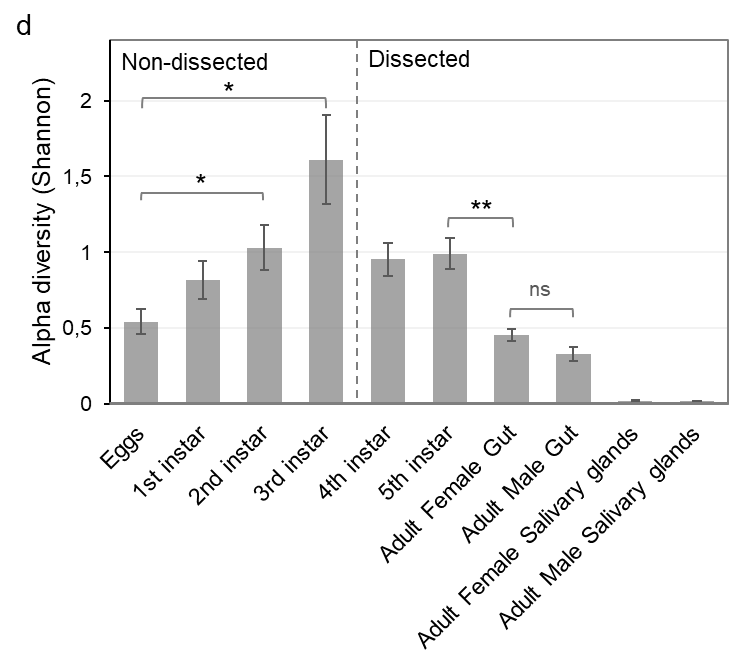
**

**
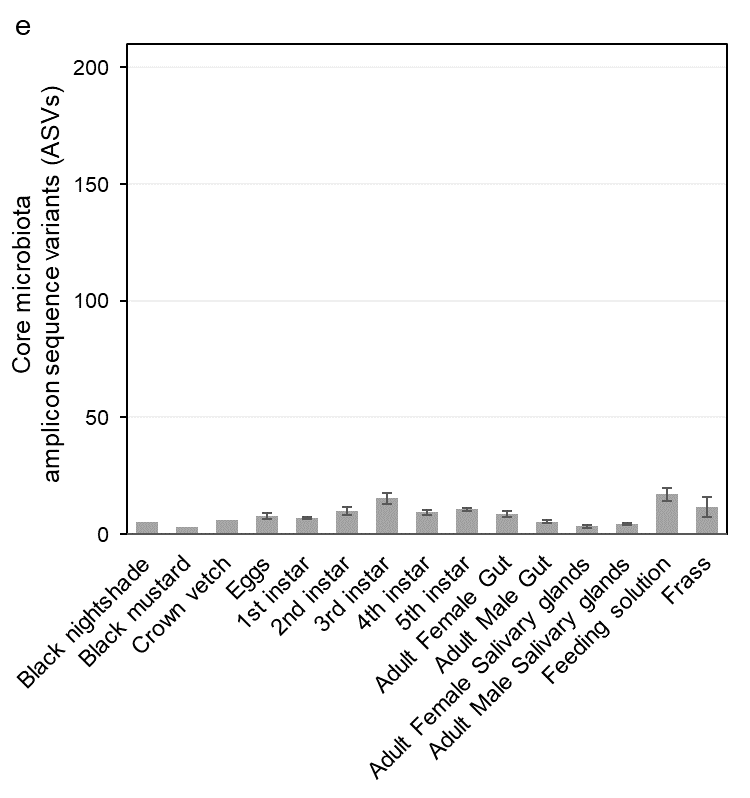
**

**
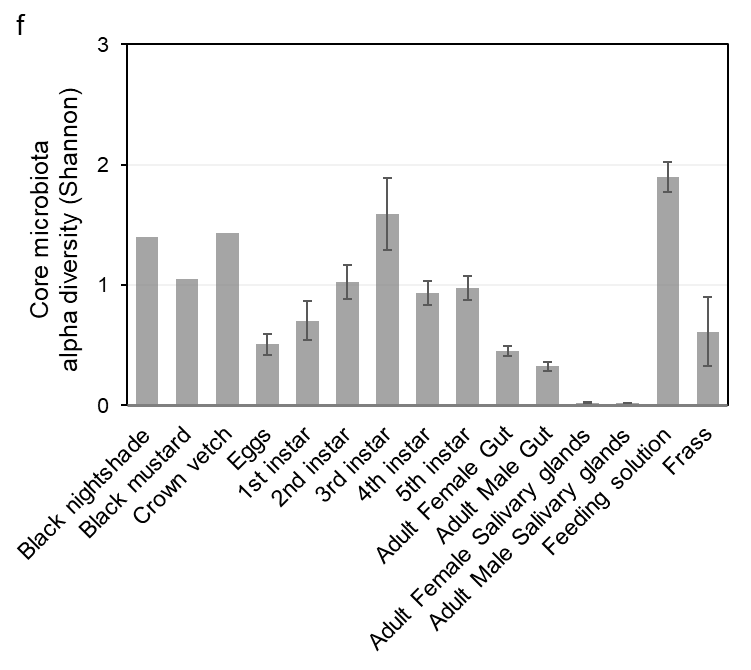

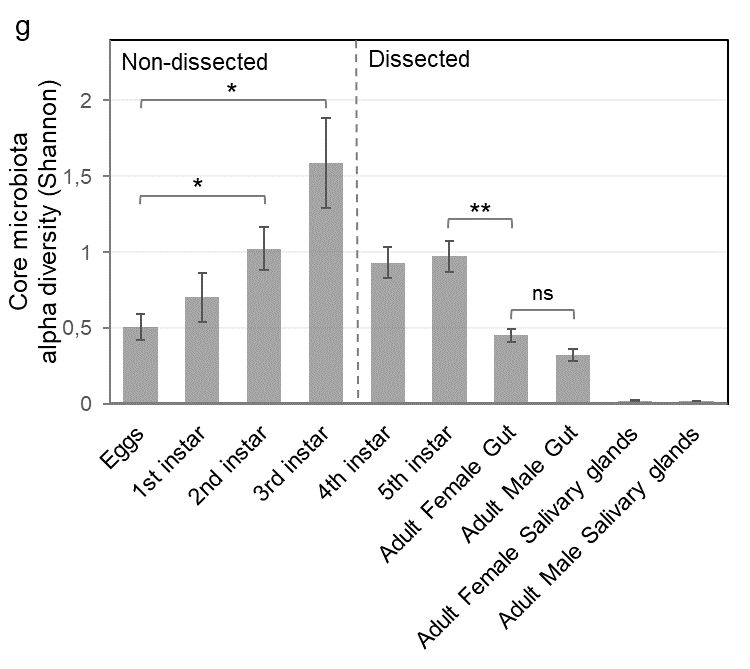
**

**Figure S3. 16S rRNA gene sequencing results and alpha diversity.** (**a**) Average number of reads for each set of 3 biological replicates for *N. viridula* samples and one biological replicate for host plant samples. (**b**) Average number of amplicon sequencing variants (ASVs) for all samples. (**c,d**) Average (Shannon) alpha diversity of all ASVs. (**e**,) Average number of ASVs for the core microbiota. (**f, g**) (Shannon) alpha diversity for the core microbiota. Error bars represent the standard error of the mean, where a Student’s *t*-test indicates significant differences (non-significant, ns; **p*≤0.05, ***p* ≤0.01).

**
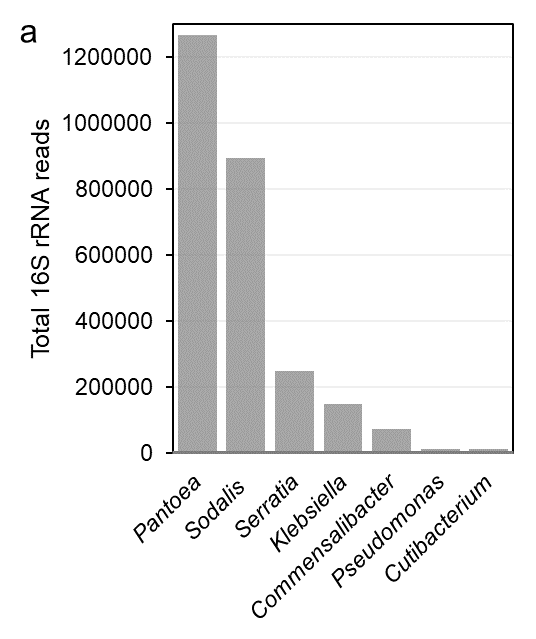

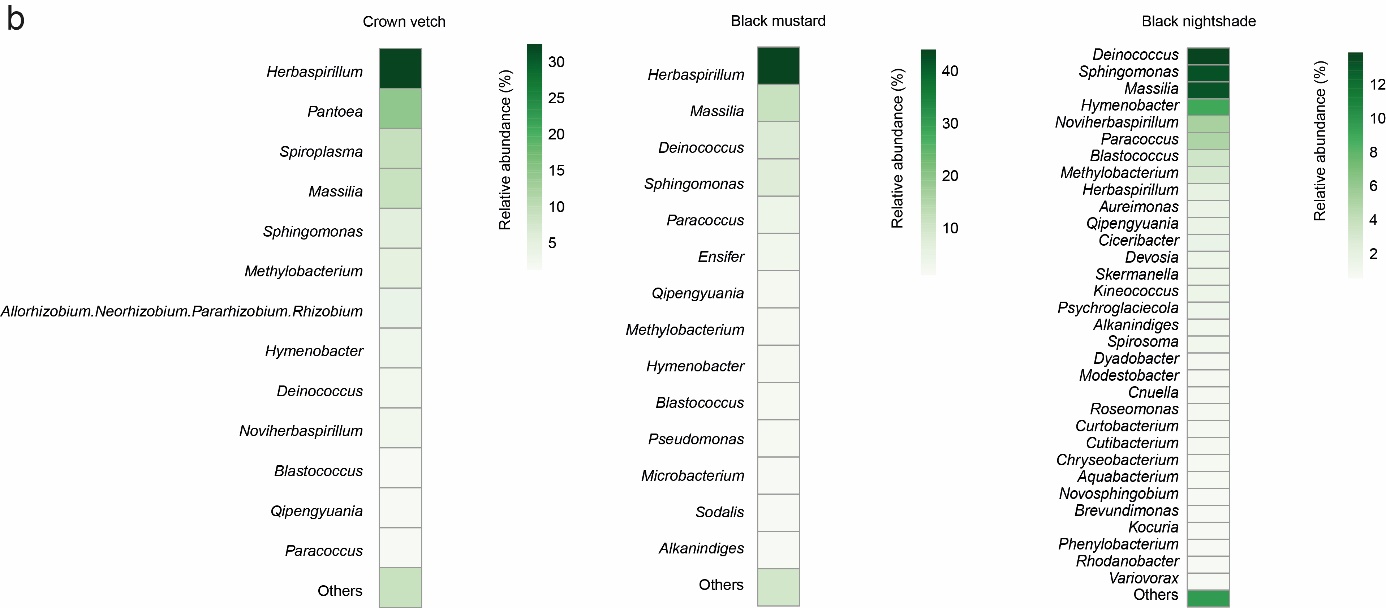
**

**Figure S4. Core microbiota of *N. viridula* and plant samples.** (**a**) Bar-graph displaying the most abundant reads in all 36 insect-associated samples (*n*=3, >10,000 reads). On the y-axis, the sum of all 16S rRNA gene reads within insect-related samples is shown and on the x-axis the genera to which these reads belong. (**b**) A heatmap showing the relative abundance (in percentage) of the top 90% of most abundant microbial genera for all three plant samples.


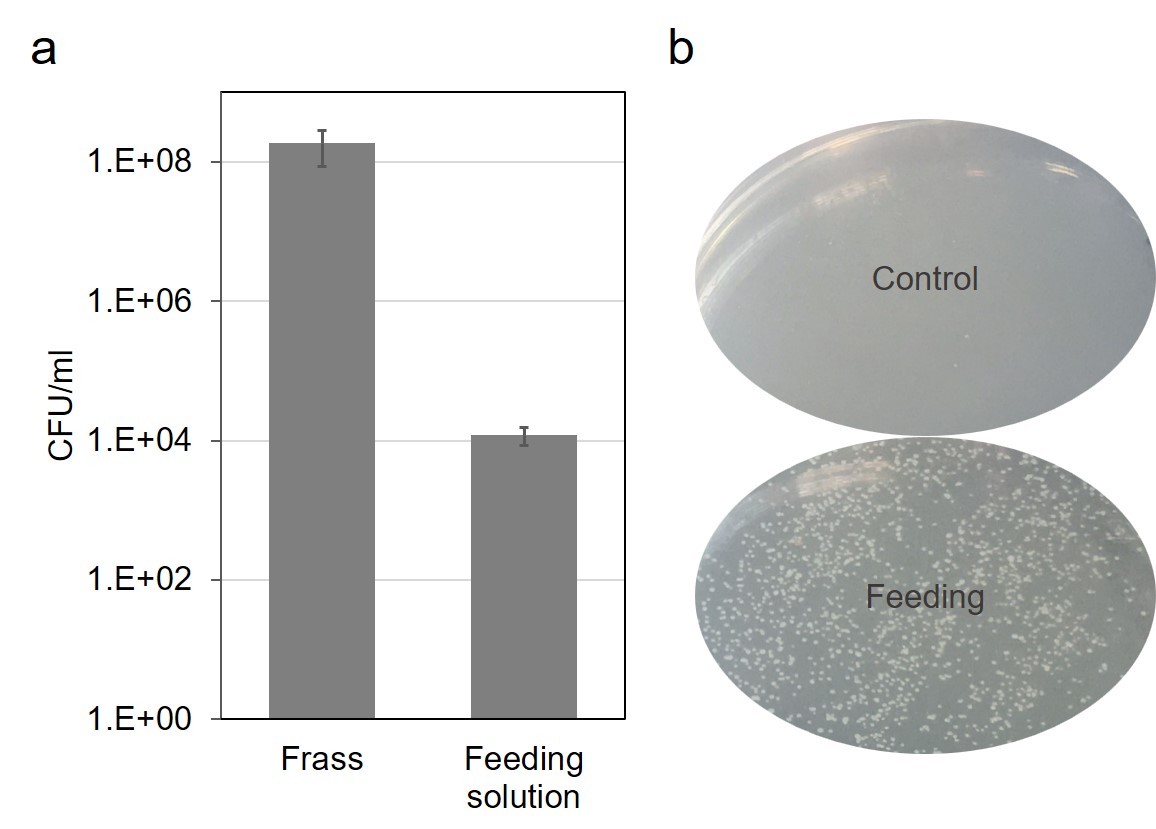


**Figure S5. Colony-forming units (CFU) are obtained from *N. viridula* frass and saliva.** (**a**) From frass and saliva collections (*n*=5 and *n*=9, respectively) colony forming units were determined on LB agar after incubations of 24 hours and 5 days, respectively, at room temperature. Bars show the CFU/ml and error bars represent the standard error of means (SE). (**b**) Photographic image of one of the feeding systems (either control or feeding) saliva collections on LB agar plate. The control feeding solution remained uncolonized after 5 days of incubation at room temperature, while the feeding solution that was offered to *N. viridula* showed clear white yeast colonies after 5 days of incubation.


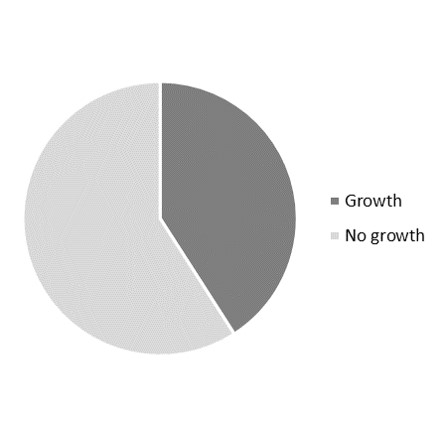


**Figure S6. Microbial growth of frass collections.** A pie chart showing the percentage of frass collections (*n* = 22) that did or did not contain visual microbiota after 24 hours of incubation on LB agar at room temperature. Each collection contained one frass droplet excreted by one insect.


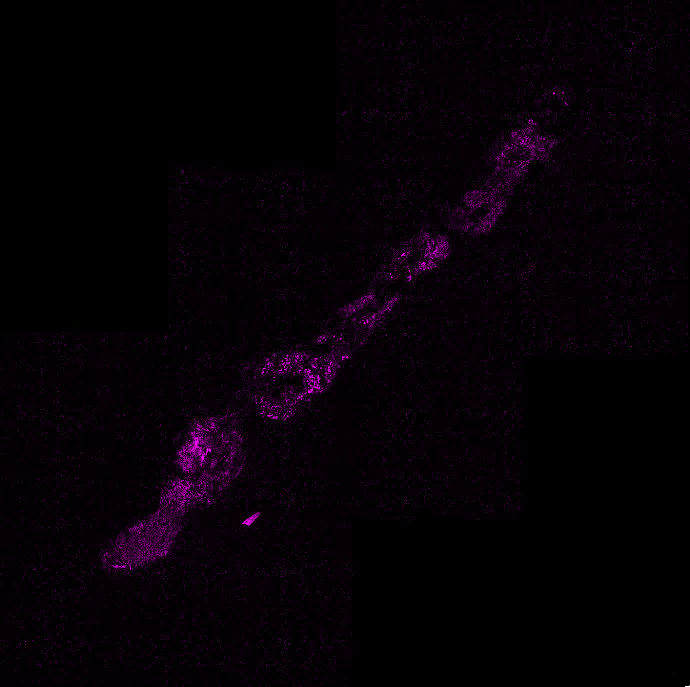
**
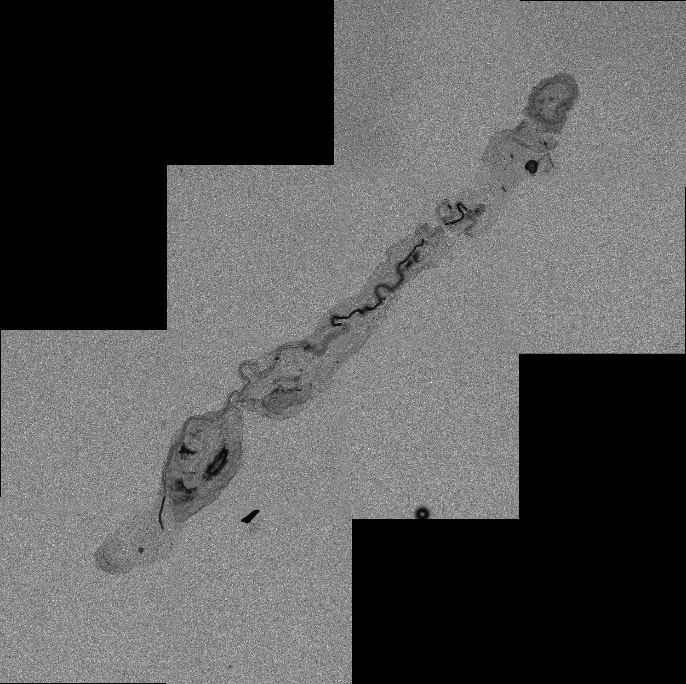
**

**Figure S7. Visualization of *Sodalis* in the salivary glands of *N. viridula*** with fluorescence *in situ* hybridization microscopy using a confocal laser scanning microscope. The micrograph showing a *N. viridula* salivary gland, with salivary duct, using FISH to visualize *Sodalis* (probe Sod1238R, Cy3, magenta) on the left image and a brightfield image in the right panel.


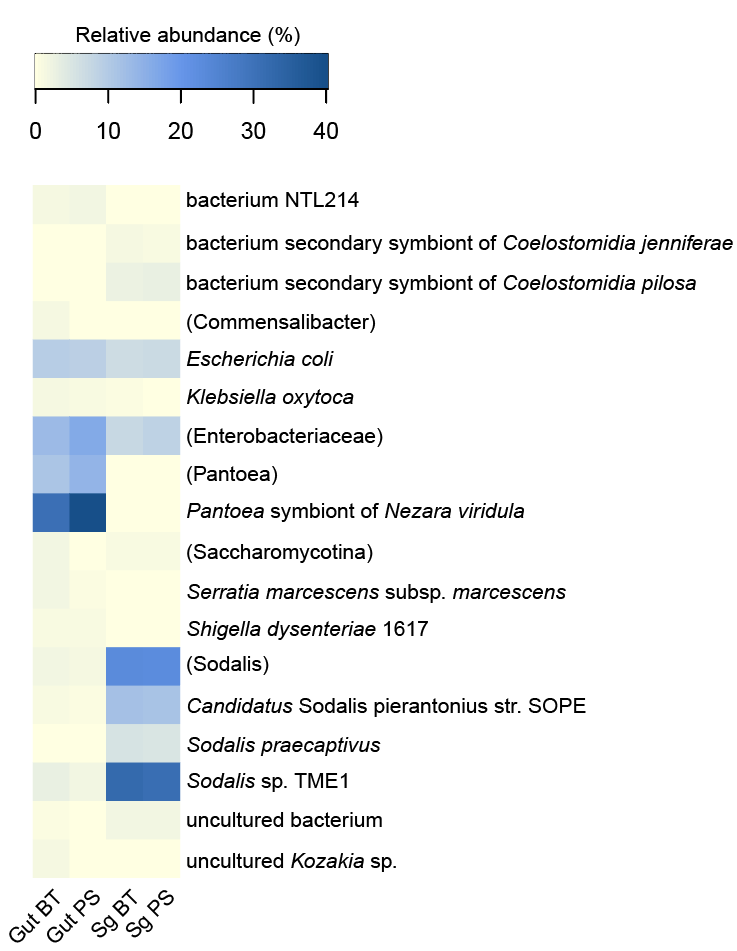


**Figure S8.** **Relative metagenome gut and salivary gland microbial abundance.** A heatmap showing the relative metagenome microbial abundance (> 1%) in adult *N. viridula* gut systems (Gut) and salivary glands (Sg), calculated percentage of reads based on raw reads from Blood&Tissue DNA isolation kit and PowerSoil kit. When the species could not be assigned, the lowest taxonomic level was shown instead and was indicated with a taxonomic name between the parentheses.

**
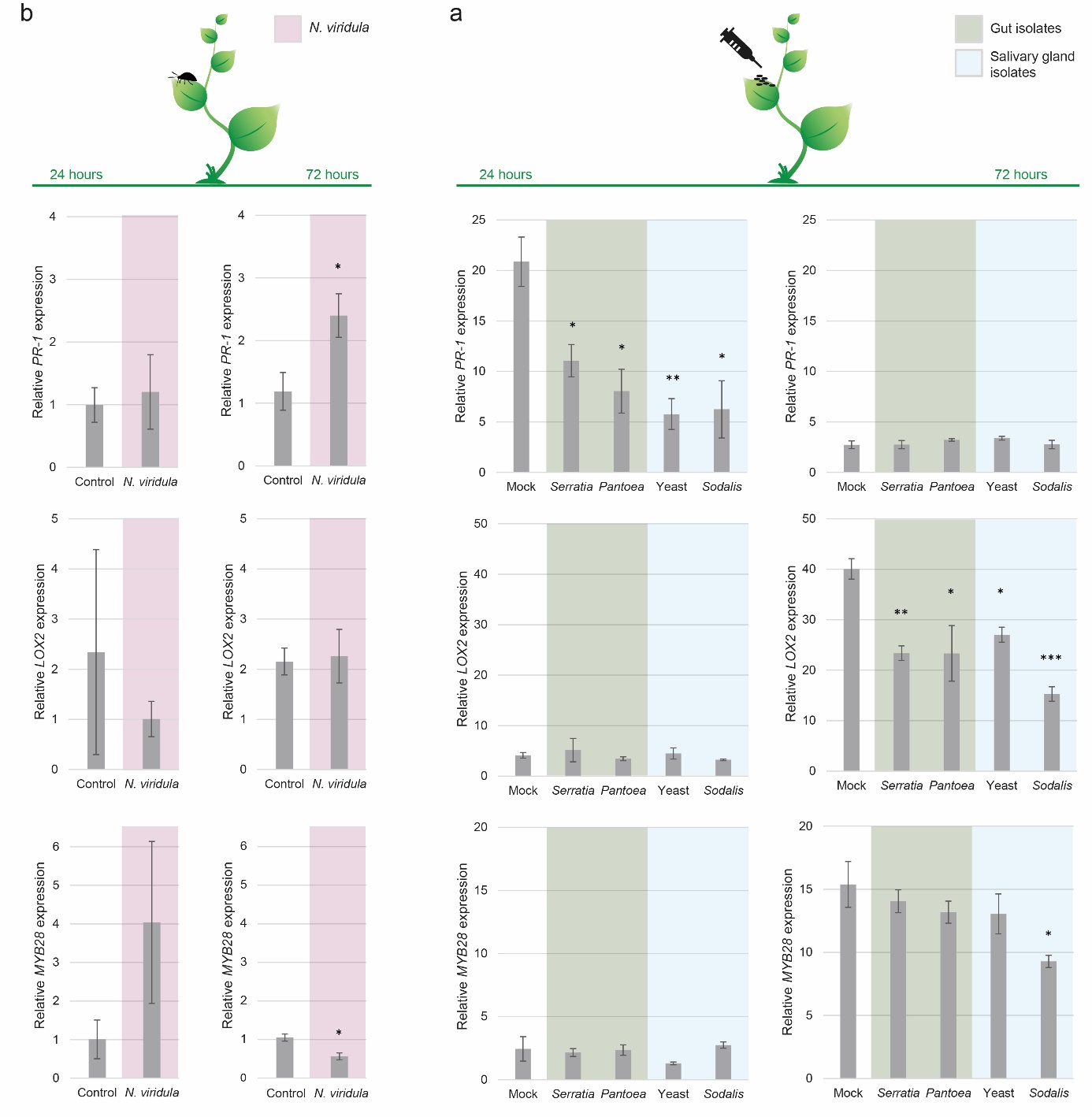
**

**b**

**a**

**Figure S9. Plant defense repression by *N. viridula*-associated microorganisms. (a)** *PR-1*, *LOX2,* and *MYB28* gene expression of local leaves (feeding damage) from 5-week-old *A. thaliana* plants infested by *N. viridula* adults for 24-72 hours and control plants without *N. viridula* (*n* = 3, 6 plants, 9 technical replicates). **(b)** *PR-1*, *LOX2,* and *MYB28* gene expression of 5-week-old *A. thaliana* plants at 24 and 72 hours after pierce-inoculation with a 5 µL droplet of 10 mM MgSO4 (*i.e.*, mock) or 1x10^8^ CFU/mL of either *Serratia*, *Pantoea*, *Yeast* or *Sodalis* in 10 mM MgSO4 (*n* = 3, 3 plants, 4 technical replicates). Bars represent the average gene expression relative to *PP2AA3* and error bars represent the standard error of means. Asterisks show Student’s *t*-test significant differences between mock and treatment (**p* ≤ 0.05, ***-p* ≤ 0.01, ****p* ≤ 0.001).

**Table S1.** Overview of the recovered MAGs. The information for each of the MAGs includes bacterial ID, genome size, GC content, completeness, contamination, number of contigs, and the relative abundance assigned based on the percentage of mapped reads. The relative abundance of unmapped reads was assigned to either mitochondrial or chloroplast reads and therefore was left out of the table.

| Bacterial IDs (genus) | Genome size (bp) | GC content (%) | Completeness (%) | Contamination (%) | Contigs (n) | Relative abundance (%)^1^ | | | |
| --- | --- | --- | --- | --- | --- | --- | --- | --- | --- |
|  |  |  |  |  |  | Gut^2^ | Gut^3^ | Salivary glands^2^ | Salivary glands^3^ |
| *Commensalibacter* | 2009072 | 37.5 | 95.8 | 0.0 | 145 | 2.5 | 0.3 | 0 | 0.0 |
| *Pantoea* | 1426114 | 40.6 | 59.3 | 0.0 | 12 | 57 | 82.6 | 0 | 0.1 |
| *Serratia* | 5249834 | 59.8 | 99.8 | 0.5 | 91 | 9 | 4.5 | 0 | 0.0 |
| *Sodalis* | 4205851 | 56.7 | 100 | 0.0 | 189 | 7.3 | 6 | 68.5 | 70 |

^1^The abundance of the mapped reads within a sample.

^2^Sample isolated using a Blood&Tissue kit.

^3^Sample isolated using a PowerSoil kit.
